# Metabolomic Effects and Their Relationship with Intracellular/Extracellular Concentrations of Casiopeinas^®^ in Triple-Negative Mesenchymal Breast Cancer

**DOI:** 10.3390/ijms26178735

**Published:** 2025-09-08

**Authors:** Karen Resendiz-Acevedo, Martha E. García-Aguilera, Araceli Tovar-Tovar, Nuria Esturau-Escofet, Lena Ruiz-Azuara

**Affiliations:** 1 Facultad de Química, Universidad Nacional Autónoma de México, Av. Universidad 3000, Circuito Exterior s/n, CU, Ciudad de México C.P. 04510, Mexico; 2 Instituto de Química, Universidad Nacional Autónoma de México, Av. Universidad 3000, Circuito Exterior s/n, CU, Ciudad de México C.P. 04510, Mexico; 3 Edificio H Mario Molina, Circuito Mario de la Cueva, Esquina Circuito de la Investigación Científica, CU, Ciudad de México C.P. 04510, Mexico

**Keywords:** metallodrugs, Casiopeinas^®^, copper quantification, triple-negative breast cancer, metabolomics, ^1^H-NMR, ICP-MS

## Abstract

Metal-based compounds, known as metallodrugs, offer promising alternatives for cancers with limited treatment options, such as triple-negative breast cancer (TNBC). Casiopeinas^®^, a family of copper-based compounds, have shown anticancer and antiproliferative effects both in vitro and in vivo. However, their long-term effects, particularly on metabolic pathways related to invasion and metastasis, remain unknown. This study addresses the sustained impact of Casiopeina IIIia (CasIIIia) and Casiopeina IIgly (CasIIgly) on TNBC cell metabolism, as well as their relationship with intra- and extracellular copper concentrations associated with these compounds. Our results revealed effects on several pathways, including those related to amino acid, lipid, and carbohydrate metabolism and energy production, all of which are involved in epithelial–mesenchymal transition (EMT), invasion, and metastasis. These metabolic changes were accompanied by progressive intracellular accumulation of Casiopeinas^®^, suggesting a relationship between the concentration and their metabolic effects. These findings suggest that Casiopeinas^®^ could be a promising therapeutic option for TNBC treatment.

## 1. Introduction

Nowadays, metallodrugs offer a versatile alternative to traditional treatments for various diseases, thanks to the modulation of their physicochemical and stereochemical properties. Including metal ions in their structure enables distinct biological mechanisms of action, facilitates interactions with a wide range of biomolecules, and provides a potential strategy to overcome drug resistance and toxicity [1,2,3].

Since the introduction of cisplatin as a standard treatment for various types of cancer, the opportunity to study other metallodrugs has emerged [4,5]. Metallodrugs exhibit biological activity against cancer and are being studied for their effects on processes such as metastasis and invasion [6,7]. In fact, it has been reported that metallodrugs based on ruthenium, zinc, and copper, among others, can reduce these processes, although their mechanisms have not yet been fully clarified [8,9].

Understanding how metallodrugs interact with cancer cells is crucial, as these cells develop specific characteristics to adapt and ensure their survival. These characteristics, known as the hallmarks of cancer, distinguish malignant from nonmalignant cells [10,11]. Currently recognized hallmarks of cancer include sustaining proliferative signaling, evading growth suppressors, avoiding immune destruction, enabling replicative immortality, activating invasion and metastasis, inducing angiogenesis, resisting cell death, genomic instability, tumor-promoting inflammation, unlocking phenotypic plasticity, non-mutational epigenetic reprogramming, senescent cells, polymorphic microbiomes, and reprogramming energy metabolism [12,13,14].

Reprogramming energy metabolism has been of special interest because cancer cells require it to maintain nutrients, biological functions, macromolecular components, and bioenergetic demands of adenosine triphosphate (ATP) and nicotinamide adenine dinucleotide phosphate (NADPH) production, as well as proliferation and migration phenomena [15,16]. Some reprogrammed metabolic pathways are glycolysis, glutaminolysis, fatty acid, and nucleotide biosynthesis [17,18,19]. Therefore, understanding cancer cell metabolism is crucial when pursuing effective cancer therapies.

TNBC is a highly heterogeneous and aggressive form of breast cancer, lacking estrogen and progesterone receptors as well as human epidermal growth factor receptor 2 (HER2) [20,21,22]. Within the TNBC classification, distinct subtypes include basal-like 1 (BL1), basal-like 2 (BL2), mesenchymal-like (M), and luminal androgen receptor (LAR) [23,24,25]. The MDA-MB-231 breast adenocarcinoma cell line belongs to the mesenchymal-like (M) subtype [26] and is characterized by undergoing epithelial–mesenchymal transition (EMT). EMT involves the biochemical transformation of epithelial cells into mesenchymal cells, granting them enhanced migratory capacity, invasiveness, and apoptosis resistance, among other traits [27,28]. Consequently, it promotes processes such as metastasis, invasion, angiogenesis, and tumor perfusion [29,30], leading to a poor prognosis and imposing limitations on current treatments [31,32]. Likewise, metabolic deregulation has been associated with these processes [33,34].

In TNBC, elevated glucose uptake, increased lactate secretion, and alterations in enzymes, glycolytic transporters, and tumor microenvironment acidification have been observed [35,36]. This metabolic reprogramming can lead to the development of myeloid-derived suppressor cells and the creation of an immunosuppressive microenvironment [37]. On the other hand, glutaminolysis plays a crucial role in providing TNBC with biological intermediates for protein and lipid biosynthesis, nitrogen formation, and fueling the citric acid cycle (TCA cycle), which contributes to glutathione (GSH) generation, among other essential processes [19,38,39]. Lastly, lipid metabolism is utilized by TBNC for energy generation, involving increased fatty acid oxidation (FAO), which is related to metastasis processes, membrane biogenesis, energetic equilibrium, and cellular viability [33,40].

Anticancer drugs may be exploited to develop less toxic and more effective alternatives to TNBC metabolic vulnerabilities. Moreover, it is important to emphasize that these effects are closely related to intracellular drug concentration, which plays a crucial role in determining biological activity, efficacy, toxicity, and pharmacological interactions [40,41].

In this way, Casiopeinas^®^ are a coordination compound family representing a potential option in cancer treatment. Their structure features copper as the central metal, bipyridine or phenanthroline as the primary ligand, and glycinate or acetylacetonate as the secondary ligand [42,43]. Biological activity has been reported in vitro and in vivo [44,45,46,47], demonstrating selectivity for cancer cells over healthy cells [48,49]. The principal action mechanisms of Casiopeinas^®^ involve interaction with DNA [50,51,52,53], generation of reactive oxygen species (ROS), leading to GSH depletion after 6 h of drug exposure [54,55,56], and the induction of apoptosis [45,57,58].

Recently, the effect of Casiopeinas^®^ on mitochondrial function and cellular metabolism has been reported. It was observed that the compounds can interrupt the mitochondrial membrane with only 4 h of treatment [49]. In addition, CasIIgly inhibits hexokinase II activity (an enzyme responsible for regulating the first step of glycolysis) and reduces metabolic energetic metabolism after 24 h of treatment [59]. A microarray study employing CasIIgly in HeLa cells (human cervical adenocarcinoma) revealed deregulation in mitochondrial function and perturbations in the phosphatidylinositol 3-kinase (PI3K)/protein kinase B (AKT)/mammalian target of rapamycin (mTOR) pathway (PI3K/AKT/mTOR pathway) [60], which is implicated in numerous cellular processes, for instance, proliferation, invasion, migration, apoptosis, glucose metabolism, and DNA repair, among others [60,61].

Ultimately, the evaluation of Casiopeinas^®^’ biological activity against TNBC has begun. The inhibitory effects of CasIIIia and CasIIgly on TNBC cell migration and metastatic processes have been studied, revealing a reduction in both [62,63]. Additionally, the activity of encapsulated CasIIIia against this type of cancer has been assessed, showing promising efficacy with low toxicity [63].

The effect of CasIIgly on TNBC cellular cancer metabolism was also studied at two short evaluation times (20 and 40 min), revealing alterations in glycolysis, the pentose phosphate pathway, the Krebs cycle, β-oxidation, the electron transport chain, and glutaminolysis, among others [64].

Previous studies suggest that Casiopeinas^®^ present biological activity even at short exposure times with a significant effect on the metabolism of TNBC cells. However, their effect on metabolic pathways at exposure times longer than 40 min (where the effects are already noticeable) but before cell apoptosis occurs is unknown. Long-term studies are essential to elucidate the biological impact of Casiopeinas^®^ on cell metabolism and could help to evaluate their potential role in modulating processes such as EMT, invasion, migration, and metastasis, facilitating the rational design of novel coordination compounds with improved selectivity and therapeutic potential.

Furthermore, as CasIIIia is undergoing a Phase I clinical trial in Mexico, our study suggests valuable insights that may support future clinical applications and personalized treatment approaches for TNBC and potentially other malignancies.

In addition, it has not been reported whether the metabolic effects promoted by Casiopeinas^®^ are proportional to their intracellular and extracellular concentrations. It is only known that these compounds can cross the cell membrane through passive transport, facilitated by the primary ligands, resulting in their hydrophobicity [46,65].

**Figure 1:**
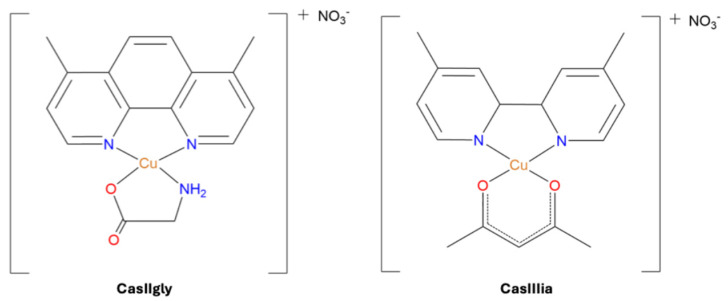
Structure of CasIIIia [Cu 4,4-dimethyl, 2,2′-bipyridine acetylacetonate] NO_3_ and CasIIgly [Cu 4,7-dimethyl, 1,10-phenanthroline glycinate]NO_3_ Casiopeinas^®^ used in this study (water molecules have been eliminated for clarity).

Metabolic perturbations induced by cisplatin (control) and two Casiopeinas^®^ treatments (Figure 1), as well as their intracellular and extracellular quantification, were determined in this work, employing TNBC cells (MDA-MB-231) (Figure S1) from 20 min to 6 h. Proton nuclear magnetic resonance (^1^H-NMR) and inductively coupled plasma mass spectrometry (ICP-MS) were used for these analyses, respectively.

## 2. Results

### 2.1. Treatment of MDA-MB-21 Cells

Mean half-maximal inhibitory concentration (IC_50_) values, obtained from three independent repetitions for cisplatin and Casiopeinas^®^, and their standard deviations (SD), are presented in Table 1. Casiopeinas^®^ demonstrated better biological activity against the MDA-MB-231 cell line, with CasIIgly exhibiting the lowest IC_50_. A representative image of the cells during this assay is shown in Figure S2.

**Figure 2:**
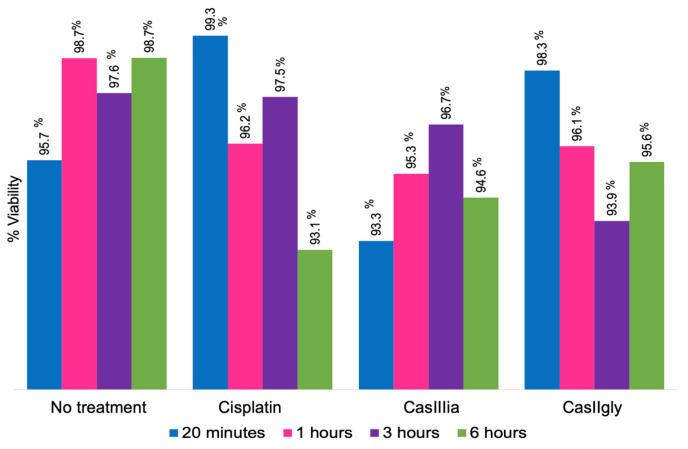
Viability percentage of MDA-MB-231 cells with the treatments at different evaluation times. The treatments were administered using the following concentrations: Cisplatin (56.25 μM), CasIIIia (26.62 μM), and CasIIgly (3.49 μM).

To verify the cells’ viability, the IC_50_ of each compound was administered to MDA-MB-231 cells at 20 min, 1, 3, and 6 h (Figure 2; Tables S1 and S2).

The complete viability data obtained are presented in Tables S1 and S2. Of note, 90% viability was observed in all cases, indicating that the cells were alive; hence, the IC_50_ concentrations were used for metabolomic experiments. The IC_50_ for each compound was used in the subsequent experiments because no significant decrease in cell viability was observed at this concentration, ensuring that cells were exposed to a sufficient compound concentration to induce significant biological activity without promoting cell death.

The times 20 min and 6 h were selected to evaluate the immediate and long-term effects of the compounds on cell metabolism. The same time points were used for intracellular concentration analysis to allow for direct comparisons.

### 2.2. Statistical Data Processing

**Figure 3:**
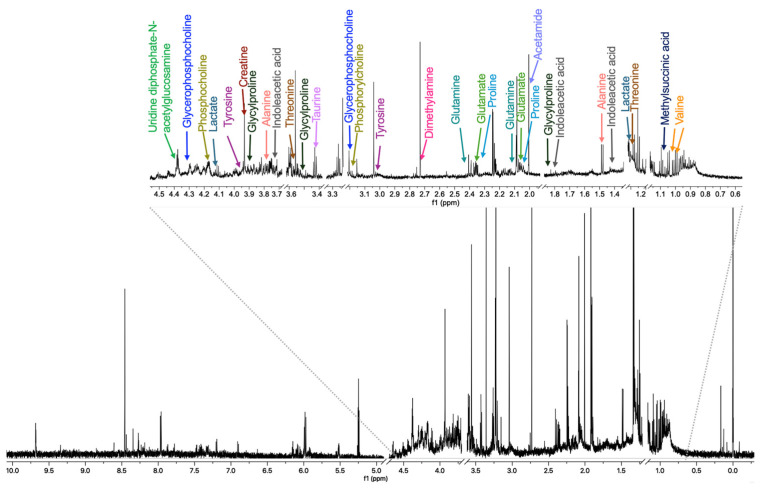
^1^H-NMR spectra (700 MHz, H_2_O/D_2_O, 300 K) of an intracellular sample from MDA-MB-231 cells treated with the CasIIgly compound at 6 h, with the assignment of some metabolites. For clarity, regions corresponding to impurities and solvent were excluded.

Once the compound concentration for cell treatments was defined, the metabolomic experiments were carried out. Figure 3 shows a representative ^1^H-NMR spectrum of an intracellular sample with some metabolites assigned. The resonance data were analyzed using chemometric methods, specifically principal component analysis (PCA), partial least squares discriminant analysis (PLS-DA), and orthogonal partial least squares discriminant analysis (OPLS-DA). These multivariate approaches were applied to determine sample variability and to identify chemical shift buckets responsible for the observed differences between sample groups.

**Figure 4:**
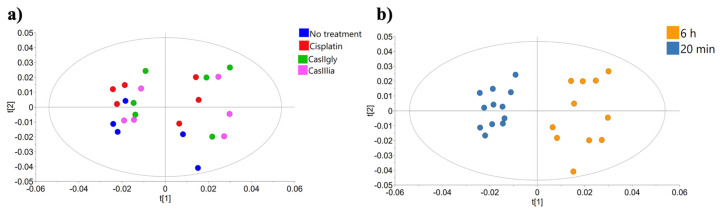
PCA scores from the NMR spectral data: (**a**) samples colored by compound; (**b**) samples colored by time.

Figure 4 shows the PCA score plots, including samples from both evaluation times. The analysis reveals a clear separation along principal component 1, driven by incubation time, with samples clustering into two groups corresponding to 20 min and 6 h, independently of the treatment applied. This pattern indicates that incubation time is the primary source of variability in the dataset. However, since the primary aim of this study is to identify metabolic pathways altered by the compounds over time, more targeted analyses were conducted to better assess treatment effects.

To this end, PCA and OPLS-DA analyses were first performed exclusively on the 20 min samples to investigate early metabolic responses. Treated samples were compared individually to untreated controls.

**Figure 5:**
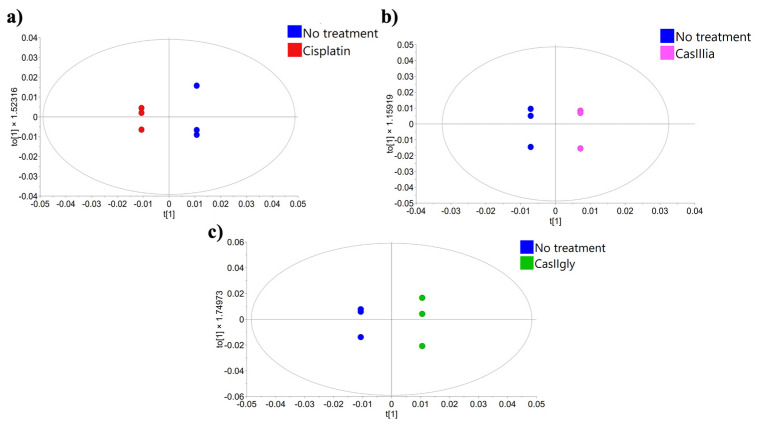
OPLS-DA scores from the NMR spectral data of 20 min samples, comparing untreated and treated groups: (**a**) no treatment vs. cisplatin; (**b**) no treatment vs. CasIIIia; (**c**) no treatment vs. CasIIgly.

Figure 5 displays the resulting OPLS-DA models, which revealed a clear separation between treated and untreated samples, suggesting that the three compounds may induce early metabolic changes.

**Figure 6:**
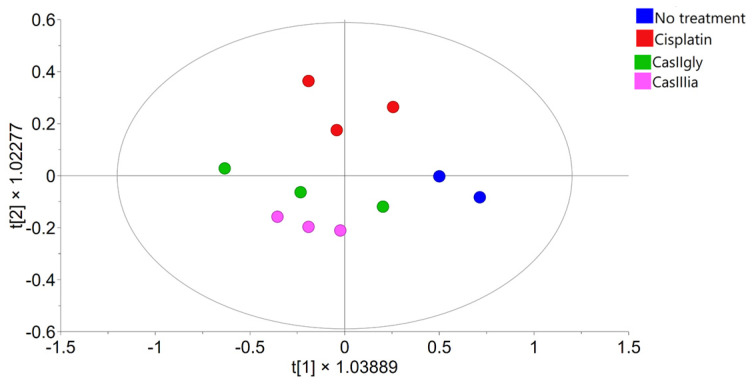
OPLS-DA scores from the NMR spectral data of 6 h samples according to treatment.

Subsequently, OPLS-DA was conducted on the 6 h samples (Figure 6), under the assumption that metabolic differences would be more evident at this later time point. The resulting model revealed a clear separation between treatment groups, indicating that the metabolic alterations induced by each compound become more pronounced over time. OPLS-DA score plots for samples collected at different treatment times supporting this approach are provided in the Supplementary Materials (Figure S3).

**Figure 7:**
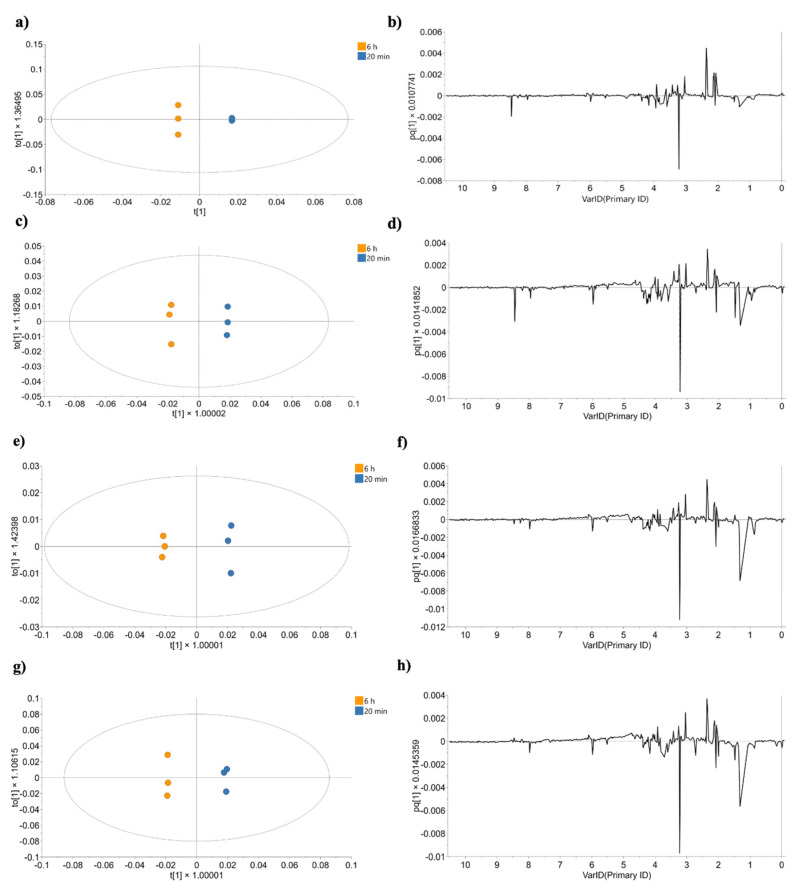
OPLS-DA scores and corresponding loading line plots from the NMR spectral data, comparing samples at 20 min and 6 h for each condition: (**a**,**b**) no treatment; (**c**,**d**) cisplatin-treated; (**e**,**f**) CasIIIia-treated; (**g**,**h**) CasIIgly-treated.

To further explore temporal effects, individual OPLS-DA analyses were conducted to directly compare the 20 min and 6 h samples for each treatment. Figure 7 shows these models, revealing a consistent separation between time points along principal component 1, as well as the corresponding loading plots that highlight the chemical shift buckets contributing to this variation.

### 2.3. Metabolite Identification and Quantification

Afterward, the chemical shift buckets responsible for sample differentiation were determined through the combined analysis of loading plots and Variable Importance in Projection (VIP) scores. This robust selection considered both the contribution of each chemical shift bucket to group separation and its statistical significance within the model.

Based on these results, the corresponding metabolites associated with the chemical shift buckets were identified and quantified. In the 20 min analysis, a total of fifteen metabolites were detected for cisplatin-treated samples, sixteen for CasIIIia-treated samples, and seventeen for CasIIgly-treated samples (Tables S3–S5). For the analysis until 6 h, nineteen metabolites were found in no-treatment samples, twenty-two were found in the cisplatin-treated samples, twenty were found in the CasIIIia-treated samples, and twenty-three were found in the CasIIgly-treated samples (Tables S9–S12). The mean concentrations from three independent assays for both analyses are summarized in the Supplementary Materials (Tables S6–S8 and S13–S16).

### 2.4. Analysis of Altered Pathways

To determine the metabolic pathways affected by treatment, pathway enrichment analysis was used, considering a *p*-value < 0.05. Nevertheless, our discussion emphasizes pathways with relevance to tumor progression and those implicated in EMT, metastasis, migration, and cell invasion.

#### 2.4.1. Analysis of Effects up to 20 min Post-Treatment

At this initial evaluation time, metabolic perturbations induced by each compound were assessed through direct comparison with untreated control cells.

Cisplatin, CasIIIia, and CasIIgly affected multiple pathways, including pyruvate metabolism, gluconeogenesis, and the Warburg effect (Table 2 and Tables S17–S19).

#### 2.4.2. Analysis of Effects up to 6 h Post-Treatment

To gain a broader understanding of how these perturbations evolve over time, metabolic pathway analysis was realized between 20 min and 6 h post-treatment. This longer-term evaluation provides insights into the sustained and cumulative effects of the compounds on cellular metabolism.

In samples without treatment, no statistically significant pathways were identified, indicating that in this case, MDA-MB-231 cell metabolism was not altered during the experiments (Table S20).

Cisplatin affected several pathways, some of which are tyrosine, cysteine, lysine, and histidine metabolism (Table S21). The metabolic changes promoted by CasIIIia included phospholipid and phosphatidylcholine biosynthesis, the citric acid cycle, sphingolipid metabolism, ketone body metabolism, and the mitochondrial electron transport chain (Table S22). In contrast, CasIIgly affected a broader range of metabolic pathways compared to CasIIIia, including phospholipid biosynthesis, glutathione metabolism, phenylacetate metabolism, and amino acid metabolism (Table S23). Overall, CasIIgly exhibited more pronounced metabolic effects, particularly evident at the 6 h time point. All of these results are summarized in Table 3.

### 2.5. Quantification of Copper Associated with Casiopeinas^®^

The quantified concentration of copper is presented in Supplementary Materials Tables S25 and S26. Table 4 presents the mean copper concentration in MDA-MB-231 cells, showing the standard deviations, for intracellular and extracellular samples, with and without Casiopeinas^®^ treatment. It is important to note that consequently, copper concentrations can differ in cells due to factors such as differences in proliferation and cell cycle phases.

In the control samples, intracellular copper levels decrease from 20 min to 6 h, while those of extracellular copper increase. This could be caused by homeostasis processes occurring within the cells. In contrast, with Casiopeinas^®^, intracellular copper concentrations increase, while extracellular levels decrease from 20 min to 6 h, suggesting that the compound may be entering the cells. After 6 h of treatment, the intracellular concentration was higher than at 20 min. Moreover, when using CasIIgly at 6 h, the amount of copper outside the cells could not be detected.

To measure copper concentrations (Table 4), the basal copper was subtracted to consider only the amount of copper associated with Casiopeinas^®^. Table 5 presents the initial copper concentrations in the doses used for CasIIIia and CasIIgly and their concentrations in the intracellular and extracellular compartments.

**Figure 8:**
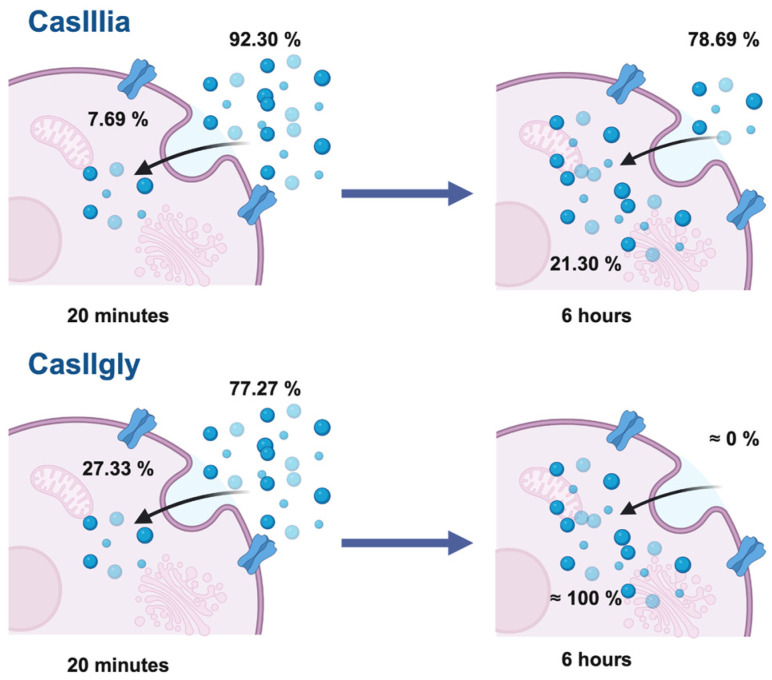
Estimated percentage distribution of CasIIIia and CasIIgly between intra- and extracellular compartments at both treatment times.

The estimated distribution percentages of Casiopeinas^®^ in MDA-MB-231 cells are presented in Figure 8, where it is observed that the entry of CasIIgly occurs rapidly. After 20 min, almost 30% of the total compound concentration is found inside the cell, and after 6 h of treatment, the compound may be entirely inside the cell. In contrast, CasIIIia has only entered 20% of its total concentration after 6 h.

## 3. Discussion

Cisplatin showed an impact on pathways such as pyruvate metabolism, gluconeogenesis, and the Warburg effect in the first 20 min of exposure. After 6 h, it perturbed the metabolism of various amino acids. Cisplatin mainly exerts its antitumor effects through the formation of DNA adducts, leading to replication stress and activation of the DNA damage response. However, some metabolic perturbations were detected, which could be indirectly attributed to secondary effects associated with its mechanism of action. In particular, the generation of ROS and mitochondrial dysfunction, both well-documented consequences of cisplatin exposure, may contribute to alterations in pathways related to energy metabolism and redox balance [66]. Thus, although metabolic pathways are not the primary targets of cisplatin, the observed changes likely reflect cellular adaptations to DNA damage and oxidative stress induced by the treatment. Such metabolic alterations have been previously described as secondary responses to genotoxic stress [67,68]. Given these considerations, the lack of direct metabolic targeting underscores the need to explore new therapeutic agents capable of exerting a primary and specific disruption of tumor metabolism, particularly in cancers that depend on metabolic reprogramming for survival and progression.

On the other hand, the metabolic analysis at 20 min confirmed that the biological activity of CasIIIia and CasIIgly on cancer cell metabolism occurs rapidly. The observed effects suggest that carbohydrate metabolism is one of the early targets of Casiopeinas^®^, which are particularly relevant in cancer cell metabolism, because of its relation to invasion, metastasis, migration, and chemoresistance processes [69].

Aerobic glycolysis redirects pyruvate to lactate formation, even under normoxia, known as the Warburg effect, enhancing EMT and invasive phenotypes [70]. By diverting pyruvate from mitochondrial oxidation, pyruvate dehydrogenase is inhibited, and chemoresistance and EMT are promoted [71]. Meanwhile, citric acid cycle intermediates fuel anabolic reactions and epigenetic modifications of EMT regulators [72,73].

In particular, TNBC is highly dependent on glucose metabolism to meet its energy demands. Inhibition of this pathway impairs metastasis and migration by reducing cell contractility, a key factor in cell division and motility [72,73]. This work demonstrates that Casiopeinas^®^ exert early interference with carbohydrate metabolism, particularly glucose-related pathways, aligning with emerging therapeutic strategies that target these routes to limit cancer cell adaptation, invasiveness, and dissemination [74].

The analysis performed from 20 min to 6 h allowed for the identification of additional metabolic targets of Casiopeinas^®^, one of which is lipid metabolism. Many metabolic pathways related to lipid metabolism have been associated with EMT, invasion, and metastasis. Sphingolipid metabolism plays an important role in cancer progression, where metabolites such as sphingosine-1-phosphate are crucial in cellular motility through signaling cascades, thus contributing to EMT. Phospholipid and phosphatidylcholine biosynthesis favors cell membrane modification, required for cellular migration, providing energy, membrane structural components, and lipid signaling for cellular plasticity regulation [75]. β-hydroxybutyrate, an intermediate in ketone body metabolism, serves as an energy alternative source and regulates epigenetic functions to maintain the plasticity of cancer cells under metabolic stress, increasing their metastatic potential [39]. The results of this work are consistent with previous studies identifying lipid metabolism as a driver of cell plasticity and invasion in TNBC [76], reinforcing the idea that Casiopeinas^®^ interfere with the maintenance of metastatic phenotypes.

Amino acid metabolism affected by CasIIgly is essential for maintaining cellular homeostasis and acts as a metabolic regulator for cancer cell growth, modulating cellular motility as well as invasiveness and metastatic potential [77], also participating in EMT. Nevertheless, it is not yet clear how this route contributes to cell conversion [70]. One important amino acid is glutamine, the most abundant in the human body, which performs several biosynthetic and cellular processes. Its metabolization leads to the formation of glutamate and GSH, both involved in redox balance, and is used as a nitrogen and carbon source for synthesizing nucleotides and non-essential amino acids. Furthermore, they are important in signal transduction in cancer cells [13]. The observed amino acid disruptions further support the role of these compounds in cancer cell viability and redox homeostasis.

Lastly, the mitochondrial electron transport chain, affected by CasIIIia, is a source of ROS responsible for TGF-β signaling, which plays an important role in regulating EMT, metastasis, and invasion, promoting cell motility and invasive capacity. In addition, it supports metastatic cells by providing ATP, necessary for sustained migration, redox balance, and mitochondrial membrane potential, enabling cancer cell survival [78]. The data point to a possible association between mitochondrial dysfunction triggered by Casiopeinas^®^ and reduced invasive potential.

The interaction between Casiopeinas^®^ and the metabolic pathways mentioned before is involved in the promotion of invasion and metastasis processes, as supported by the findings reported by González-Ballesteros, which showed that these compounds inhibited the migration and invasion capacity of TNBC cells [62]. Together, these previous findings and the results of this study suggest potential antimetastatic activity, positioning Casiopeinas^®^ as promising agents with a novel mechanism of action based on metabolic interference.

In addition to the metabolic analysis, it was observed that CasIIIia and CasIIgly induced variations in the metabolite concentrations from 20 min to 6 h, showing different metabolic profiles compared to non-treated cells. These variations reflect the different impact of each compound on metabolic processes over time, with both interfering with metabolic processes shortly after administration and CasIIgly affecting a greater number of pathways. The time-resolved nature of this analysis reveals dynamic metabolic responses, enhancing the novelty of this study compared to previous analyses based on static endpoints.

This result may be mediated by biological activity, considering that CasIIgly is almost nine times more active than CasIIIia; consequently, cellular metabolism is more compromised in the presence of CasIIgly, potentially inducing a reduction in EMT and possible cell apoptosis, which, as mentioned earlier, depend on the metabolic processes [79].

Although the metabolic effects of both Casiopeinas^®^ differ in MDA-MB-231 cells, it is evident that they can modulate cancer cell metabolism and induce significant biological activity on the crucial metabolic functions of tumor cells; this positions them as a promising alternative for the treatment of the complex profile of TNBC and its progression. In this context, this study contributes novel insights into how metallodrugs can impact tumor metabolism beyond classical cytotoxicity.

In line with previous in vitro and in vivo studies, Casiopeinas^®^ have demonstrated selectivity toward cancer cells while exhibiting low cytotoxicity in healthy cells, such as lymphocytes, compared to cisplatin [49]. The lethal dose response is 200 mg/m^2^ for CasIIIia and 160 mg/m^2^ for CasIIgly, with an elimination rate of 8 mg/kg in Wistar rats. These findings suggest that both compounds are rapidly cleared from the bloodstream, potentially reducing systemic toxicity. In addition, their therapeutic safety margins are 15.5 to 77.7 mg/m^2^ and 66.6 to 166.6 mg/m^2^, respectively [80]. Notably, selectivity may be influenced by differences in metabolic profiles between normal and cancerous cells, where the latter rely more heavily on altered metabolic pathways, such as aerobic glycolysis and fatty acid biosynthesis, which were significantly affected by Casiopeinas^®^, demonstrating the potential of these compounds to exploit metabolic vulnerabilities unique to cancer cells, thereby providing a basis for therapeutic selectivity.

This work represents one of the first metabolomic studies performed with copper coordination compounds, varying the exposure time to assess their metabolic impact. However, an aspect beyond the scope of this study is the use of a single TNBC cell line. While this model provides valuable insights into the metabolic effects of Casiopeinas^®^ to achieve a more comprehensive understanding of their selectivity and therapeutic potential, future studies should incorporate additional cancer and healthy cell lines. Nevertheless, this time-course metabolomics approach opens avenues for future mechanistic and translational research on metal-based drugs.

The ICP-MS results showed that CasIIgly crosses the cell membrane more rapidly than CasIIIia. The ability of Casiopeinas^®^ to cross the membrane is influenced by their chemical structure. The hydrophobicity of the primary ligand either facilitates or hinders cellular uptake. In the case of CasIIgly, the presence of phenanthroline promotes its easier entry, enhancing its uptake, causing copper overload in the cells, whereas CasIIIia enters the cell more gradually, resulting from the acetylacetonate ligand [17].

It was observed that the metabolic effect is related to their concentration inside the cell, which was proportional to the magnitude of activity. This is further supported by the observation that the biological activity of CasIIgly on MDA-MB-231 cells was higher (with a lower IC_50_). The metabolic impact was more pronounced at 6 h, affecting important metabolic pathways. At this time, the intracellular concentration of CasIIgly was nearly 100% of the initial concentration.

Altogether, this reinforces the connection between compound accumulation, metabolic disruption, and functional anticancer activity, highlighting the relevance of metal coordination chemistry in drug design.

## 4. Materials and Methods

### 4.1. Cell Culture

The MDA-MB-231 cell line was cultured in Dulbecco’s Modified Eagle Medium (DMEM-F12) (Biowest, Nuaillé, France), supplemented with 10% fetal bovine serum (Biowest, Nuaillé, France), 1% antibiotic-antimycotic (Biowest), and 1% nonessential amino acids (Gibco, Thermo Fisher Scientific, Waltham, MA, USA) at 37 °C in a humidified atmosphere with 5% CO_2_ until reaching 80% confluence for subsequent experiments. A representative phase-contrast image of a culture of the MDA-MB-231 cells obtained is provided in the Supplementary Materials, Figure S1.

### 4.2. Determination of the Half-Maximal Inhibitory Concentration (IC_50_)

The IC_50_ of the compounds was determined in MDA-MB-231 cells using a 3-(4,5-dimethylthiazol-2-yl)-2,5-diphenyl tetrazolium bromide (MTT) assay, as reported by Mossman [81], in 96-well plates at a concentration of 10,000 cells/well and incubated for 24 h with different concentrations of cisplatin and Casiopeinas^®^ at 37 °C, in a humidified atmosphere with 5% CO_2_. Subsequently, the MTT reagent was added to the plates and incubated for 4 h without exposure to light. To conclude, the plates were analyzed using a microplate reader (AgileReader^®^, PerkinElmer Inc., Waltham, MA, USA) at 570 nm. Representative phase-contrast images of untreated and treated MDA-MB-231 cells obtained during the IC_50_ assays are provided in the Supplementary Materials, Figure S2.

### 4.3. Viability Assays

Cell viability was verified in triplicate for different times, including the evaluation times (20 min, 1, 3, and 6 h) for untreated cells and cells treated with the compounds, using the IC_50_ determined for each.

The trypan blue method (4%) was employed to assess viability, utilizing a Neubauer camera for counting dead, live, and total cells. Viability was calculated by taking the ratio of live cells to total cells and multiplying by one hundred [82].

### 4.4. Intracellular Metabolite Extraction

In metabolomic experiments, cells were incubated in triplicate for 20 min and 6 h in 10 mm Petri dishes, with and without treatment. The compounds were administered to correspond to the determined IC_50_ values.

Following the incubation period, the growth medium was collected and saved, and the cells were washed three times with 1 mL phosphate buffer (Gibco, Thermo Fisher Scientific, Waltham, MA, USA), scraped, and counted using the trypan blue assay. Subsequently, the cell samples were centrifuged at 3500× *g* for 5 min at 4 °C, and the supernatant was recovered and kept on ice for 5 min. Later, 1 mL of the acetonitrile–water mixture (50%) was added. At the final stage, the intracellular samples were sonicated for 20 min, followed by centrifugation at 14,500× *g* for 15 min at 4 °C. The supernatant was recovered, and the acetonitrile was evaporated [83]. The dried metabolite samples were resuspended in 700 μL of PBS-TSP solution (NaH_2_PO_4_ (1 mM)/Na_2_HPO_4_ (1 mM)/TSP (0.02 mM), pH = 7.4). Subsequently, they were homogenized and centrifuged at 12,000× *g* for 5 min. The supernatant of each sample was transferred to a 5 mm NMR tube for its analysis.

### 4.5. ^1^H-NMR Analysis

NMR spectra were collected at 298 K on an Avance III HD spectrometer of 16.4 T, operating at a ^1^H frequency of 699.95 MHz, equipped with a 5 mm z-axis gradient TCI cryoprobe and SampleJet automatic sample changer. ^1^H-NMR spectra were acquired with the standard NOESY-ID pulse sequence (noesypr1d). Water resonance was irradiated during a relaxation delay of 4.0 s and during the mixing time of 10 ms; a total of 128 scans were collected into 64 K data points using a spectral width of 14 kHz and an acquisition time of 2.3 s. An exponential line broadening factor of 0.3 Hz was applied to the free induction decay before the Fourier transformation. All spectra were manually processed by phase, baseline corrected, and referenced internally to Sodium 3-(trimethylsilyl) (^2^H_4_)propanoate (TSP) signal (0.00 ppm) using TopSpin 3.5 pl 6 (Bruker, Billerica, MA, USA).

### 4.6. Statistical Analysis

Data matrices were constructed after processing the spectral samples, using Chenomx NMR Suite v. 11 (Chenomx Inc., Edmonton, AB, Canada). The matrices were divided into buckets of 0.02 ppm and normalized to the total area, excluding water, ethanol, impurities, and TSP signals. Subsequently, the data matrices underwent analysis with SIMCA 18.0.1 software (Umetrics, Sartorius Stedim Biotech, Umeå, Sweden), employing the chemometrics methods previously mentioned (PCA, PLS-DA, and OPLS-DA).

The quality of each model was evaluated using parameters like the goodness of fit (R^2^), the goodness of prediction, and the fraction of the total variation predicted by a component (Q^2^). The values of loadings and VIP scores were used to identify the buckets responsible for the sample separation.

### 4.7. Metabolite and Metabolic Pathway Identification

Metabolite identification and quantification were performed using Chenomx NMR Suite v. 11 (Chenomx Inc., Edmonton, AB, Canada), based on their chemical displacement, multiplicity, and coupling constants. TSP was used as an internal reference, considering the area under the curve. The metabolite concentrations in each sample were normalized to the number of cells quantified at the end of treatments for subsequent comparison and analysis.

Finally, the metabolic pathways altered by the treatments were analyzed with MetaboAnalyst 5.0 software through quantitative enrichment analysis (QEA), incorporating the concentrations of metabolites. For 20 min analysis, untreated and treated samples were compared. For the 20 min to 6 h analysis, samples treated with the same compound were compared over time.

The biochemical routes with a *p*-value < 0.05 were considered statistically significant. For the interpretation and discussion of the results, particular emphasis was placed on those implicated in cancer progression, EMT, metastasis, migration, and cell invasion.

### 4.8. ICP-MS Analysis

ICP-MS copper quantification was performed in a Perkin Elmer^®^NEXION 2000, equipped with a Quadrupole Ion Deflector. Before the analysis, calibration and verification were carried out using the NexIon Setup Solution (Perkin Elmer Inc., Waltham, MA, USA). System optimization included a performance check, torch alignment, nebulizer gas flow adjustment, mass calibration, and resolution verification to ensure analytical quality. Both copper isotopes, Cu^63^ and Cu^65^, were analyzed.

In 10 mm Petri dishes, 1.5 × 10^6^ cells per plate were incubated in triplicate for 20 min and 6 h, with the Casiopeinas^®^ and without treatment. The compounds were administered at concentrations corresponding to the determined IC_50_ values. Subsequently, the growth medium was collected, and the cells were washed twice with 1 mL of cold Phosphate-Buffered Saline (PBS). Then, the cells were lysed and scraped using a lysis buffer (NaCl 150 mM, EDTA 1 mM, SDS 0.1%, Triton X-100, Tris-HCl 50 mM). The lysates were recovered and centrifuged at 12,500× *g* for 15 min. The supernatant was distributed for protein determination and copper quantification assays.

Prior to the quantification assays, 500 μL of each sample was pre-digested by adding 1 mL of 69–70% suprapure HNO_3_ (J.T. Baker^®^, Avantor, Phillipsburg, NJ, USA), followed by microwave-assisted digestion for 1 h using a predetermined program (Anton Paar, Multiwave Pro, Graz, Austria). This program involved controlled temperature and pressure conditions, reaching up to 220 °C and approximately 60 bar. The resulting mixtures were then transferred to 5 mL volumetric flasks.

A calibration curve (counts per second/concentration) was constructed for mass spectra acquisition, using concentrations of 2, 3, 4, 5, 6, 7, 8, 9, 10, 15, and 20 ng/mL with a Multi-elemental High Purity Standard (HPS) ICP-MS-68B-A-100 (lot #: 2301306-100).

#### Copper Quantification

The copper concentrations in each sample were calculated using both isotopes’ relative abundance and were normalized (Supplementary Materials, Table S24). In the extracellular samples, the copper concentration in the growth medium was adjusted for a better analysis.

The copper associated with Casiopeinas^®^ was calculated by considering intracellular and extracellular copper concentrations, representing the total copper concentration. The basal copper levels in each sample were determined and subtracted from the total copper concentrations to obtain the actual amounts of copper associated with Casiopeinas^®^. These values were then compared to the copper concentration administered with the compounds at the beginning of the treatments, and the percentage distribution of Casiopeinas^®^ inside and outside the cells was estimated at two evaluation times.

## 5. Conclusions

This study demonstrates that metallodrugs called Casiopeinas^®^, specifically CasIIgly and CasIIIia, disrupt key metabolic pathways, including the Warburg effect, pyruvate metabolism, the citric acid cycle, sphingolipid metabolism, phospholipid and phosphatidylcholine biosynthesis, and the mitochondrial electron transport chain. These pathways play central roles in maintaining EMT, invasion, migration, and metastasis processes in cancer cells. Importantly, the observed metabolic effects occurred both at early and late time points, indicating that Casiopeinas^®^ exert a sustained and dynamic impact on TNBC cancer cell metabolism, with progressively increasing activity throughout the course of treatment.

In both cases, a relationship was found between inhibitory activity, metabolic alterations, and intracellular compound concentration. CasIIgly exhibited greater biological activity and accumulated at higher concentrations inside the cancer cells, attributable to its higher hydrophobicity compared to CasIIIia. This correlation between cellular uptake, metabolic disruption, and biological response reinforces the mechanistic relevance of Casiopeinas^®^ as modulators of tumor metabolism. This is supported by previous in vivo experiments, which demonstrate that CasIIgly exhibits higher activity compared to CasIIIia, suggesting that Casiopeinas^®^ could be a promising candidate for TNBC treatment, with the potential to interfere with EMT and consequently reduce invasion, migration, and metastasis.

The findings of this work provide a valuable foundation for understanding the metabolic effects of copper-based compounds in cancer. By demonstrating that Casiopeinas^®^ can directly modulate pathways critical for tumor progression, this study highlights their potential as metabolic therapeutic agents beyond conventional DNA-targeting approaches. Future research is needed to include additional cell lines, both healthy and malignant, to achieve a more comprehensive understanding of these effects.

## Figures and Tables

**Table 1:** IC_50_ values for the evaluated compounds in MDA-MB-231 cells.

Compound	IC_50_ ± SD (μM)
Cisplatin	56.25 ± 1.29
CasIIIia	26.62 ± 0.61
CasIIgly	3.49 ± 0.33

**Table 2:** Metabolic pathways altered after 20 min of treatment compared to untreated cells, relevant for EMT, metastasis, migration, and invasion processes. Red circles represent disrupted pathways; gray lines represent unaffected pathways.

	Metabolic Pathway Identified	Metabolites Identified in Samples	Cisplatin	CasIIIia	CasIIgly
Carbohydrates metabolism	Warburg effect	Lactate, glutamate, glutamine, uridine diphosphate-N-acetylglucosamine, succinate	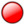	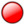	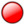
	Gluconeogenesis		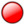	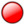	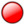
	Pyruvate metabolism		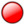	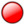	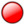
	Citric acid cycle		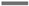	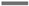	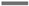

**Table 3:** Metabolic pathways altered between 20 min to 6 h post-treatment relevant for EMT, metastasis, migration, and invasion processes. Red circles represent disrupted pathways; gray lines represent unaffected pathways.

	Metabolic Pathway Identified	Metabolites Identified in Samples	Cisplatin	CasIIIia	CasIIgly
Carbohydrates metabolism	Warburg effect	Cytidine, cytosine, lactate, glucose, glutamate, glutamine, uridine diphosphate-*N*-acetylglucosamine, succinate	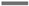	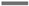	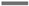
	Gluconeogenesis		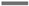	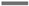	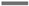
	Pyruvate metabolism		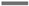	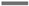	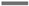
	Citric acid cycle		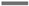	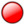	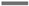
Lipids metabolism	Sphingolipid metabolism	Acetylcholine, glycerophosphocholine, phosphocholine	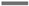	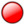	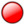
	Phospholipid biosynthesis		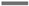	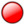	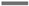
	Phosphatidylcholine biosynthesis		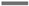	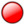	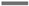
	Ketone body metabolism		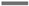	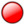	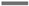
Amino acid metabolism	Arginine metabolism	Acetylcysteine, alanine, arginine, proline, glutamate, glutamine, Glycylproline, *N*-acetylglutamine, valine, taurine, threonine	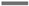	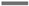	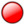
	Tyrosine metabolism		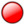	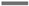	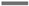
	Cysteine metabolism		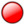	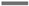	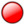
	Lysine metabolism		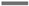	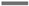	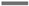
	Histidine metabolism		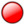	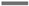	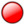
	Proline metabolism		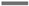	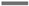	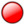
	Glutathione metabolism		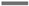	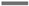	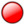
	Phenylacetate metabolism		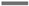	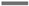	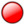
Energy metabolism	Mitochondrial electron transport chain	Creatine, creatinine, formate, NADH, phosphocreatine, succinate	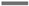	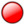	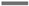

**Table 4:** Intracellular and extracellular copper concentrations in samples without and with Casiopeinas^®^ treatment at both evaluation times.

	20 min		6 h	
	Intracellular (µg Cu/mL)	Extracellular (µg Cu/mL)	Intracellular (µg Cu/mL)	Extracellular (µg Cu/mL)
Control (no treatment)	0.028 ± 0.015	0.001 ± 0.003	0.016 ± 0.002	0.008 ± 0.008
CasIIIia	0.015 ± 0.001	0.183 ± 0.008	0.032 ± 0.001	0.116 ± 0.094
CasIIgly	0.021 ± 0.004	0.007 ± 0.005	0.033 ± 0.022	^1^ ND

^1^ ND: concentration not detected.

**Table 5:** Copper concentrations associated with Casiopeinas^®^ in samples at the beginning and after each treatment time.

Compound	Evaluation Time (µg Cu/mL)	Initial Copper Concentration Administered (µg Cu/mL)	Intracellular Copper Concentration (µg Cu/mL)	Extracellular Copper Concentration (µg Cu/mL)
CasIIIia	20 min	1.69	0.13	1.56
	6 h		0.36	1.33
CasIIgly	20 min	0.22	0.17	0.05
	6 h		≈0.22	≈0

## Data Availability

The data are contained within the article and the Supplementary Materials.

## Supplementary Materials

The following supporting information can be downloaded at https://www.mdpi.com/article/10.3390/ijms26178735/s1.

## Abbreviations

The following abbreviations are used in this manuscript:

TNBC	Triple-Negative Breast Cancer
EMT	Epithelial–mesenchymal transition
CasIIIia	4,4′-dimethyl-2,2′-bipridine acetylacetonate copper(II) monohydrate nitrate
CasIIgly	4,7-dimethyl-1,10-phenanthroline glycinate copper(II) nitrate dihydrate
ATP	Adenosine triphosphate
NADPH	Nicotinamide adenine dinucleotide phosphate
HER2	Human epidermal growth factor receptor 2
BL1	Basal-like 1
BL2	Basal-like 2
M	Mesenchymal-like
LAR	Luminal androgen receptor
MDA-MB-231	Breast adenocarcinoma
TCA cycle	Citric acid cycle
GSH	Glutathione
FAO	Fatty acid oxidation
DNA	Deoxyribonucleic acid
ROS	Reactive oxygen species
HeLa	Human cervical adenocarcinoma
PI3K	Phosphatidylinositol-3-kinase
AKT	Protein kinase B
mTOR	Mammalian target of rapamycin
PI3K/AKT/mTOR pathway	Phosphatidylinositol 3-kinase/protein kinase B/mammalian target of rapamycin pathway
^1^H-NMR	Proton nuclear magnetic resonance
ICP-MS	Inductively coupled plasma mass spectrometry
IC_50_	Half-maximal inhibitory concentration
SD	Standard deviation
PCA	Principal component analysis
PLS-DA	Partial least squares discriminant analysis
OPLS-DA	Orthogonal partial least squares discriminant analysis
VIP	Variable importance in projection
DMEM-F12	Dulbecco’s Modified Eagle Medium
MTT	3-(4,5-dimethylthiazol-2-yl)-2,5-diphenyl tetrazolium bromide
TSP	Sodium 3-(trimethylsilyl)(2H4)propanoate
R^2^	Goodness of fit parameter
Q^2^	Variation predicted by a component
QEA	Quantitative enrichment analysis
PBS	Phosphate-Buffered Saline
HPS	High-Purity Standard
