# Supplementary material for: Metabolomic Effects and Their Relationship with Intracellular/Extracellular Concentrations of Casiopeinas® in Triple-Negative Mesenchymal Breast Cancer
Source: Int J Mol Sci. 2025 Sep 8;26(17):8735. doi: 10.3390/ijms26178735 (PMC12429760; doi:10.3390/ijms26178735)
Supplement: Supplementary file 1 [file ijms-26-08735-s001.zip › ijms-3740015-supplementary.pdf]

Metabolomic effects and their relationship with intracellular/extracellular Concentrations of Casiopeinas® in triple-negative mesenchymal breast cancer

Karen Resendiz-Acevedo[1], Martha E. García-Aguilera[2], Araceli Tovar-Tovar[3], Nuria Esturau-Escofet[2], Lena Ruiz-Azuara[1].

Time	No treatment	Cisplatin	CasIIIia	CasIIgly
20 minutes	96.36	100.00	95.56	100.00
	95.59	100.00	88.46	98.04
	95.12	97.96	96.00	96.88
1 hours	100.00	100.00	93.18	93.55
	96.00	96.43	94.44	96.88
	100.00	92.11	98.25	97.87
3 hours	97.73	96.30	94.29	94.44
	98.39	96.30	97.78	94.12
	96.83	100.00	98.11	93.18
6 hours	98.51	91.67	93.33	97.96
	98.67	95.24	93.33	92.31
	98.86	92.31	97.14	96.67

Table S1. Viability cellular percentage for the treatments in each repetition at evaluation times.

Time	No treatment	Cisplatin	CasIIIia	CasIIgly
20 minutes	95.69 ± 0.63	99.32 ± 1.18	93.34 ± 4.23	98.31 ± 1.58
1 hours	98.66 ± 2.31	96.18 ± 3.95	95.29 ± 2.64	96.1 ± 2.26
3 hours	97.65 ± 0.78	97.53 ± 2.14	96.73 ± 2.12	93.91 ± 0.65
6 hours	98.68 ± 0.18	93.07 ± 1.90	94.6 ± 2.20	95.64 ± 2.96

Table S2. Viability cellular percentage for the treatments in each repetition at evaluation times.

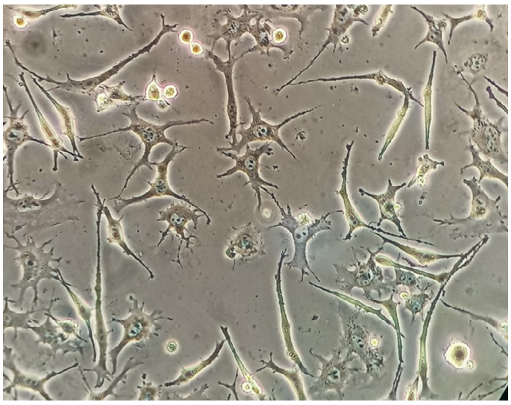

Figure S1. Representative microscopic image of MDA-MB-231 cells in logarithmic growth phase (70-80% confluence) under standard culture conditions, used to verify cell health and morphology prior to treatments. The image shows the expected spindle-shaped morphology and absence of contamination.

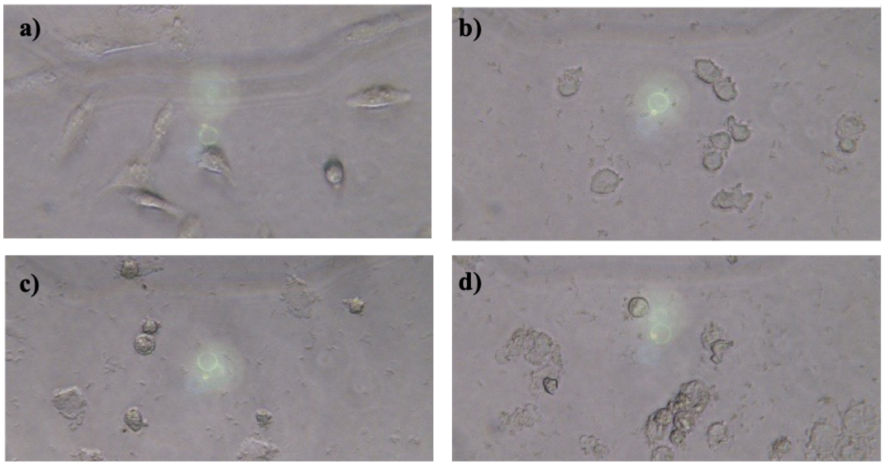

Figure S2. Representative microscopic image of MDA-MB-231 cells obtained during IC_{50} assays at 24 hours: **(a)** no treatment; **(b)** cisplatin; **(c)** CasIIIia; **(d)** CasIIgly. Images illustrate the expected morphological changes associated with reduced viability under these conditions.

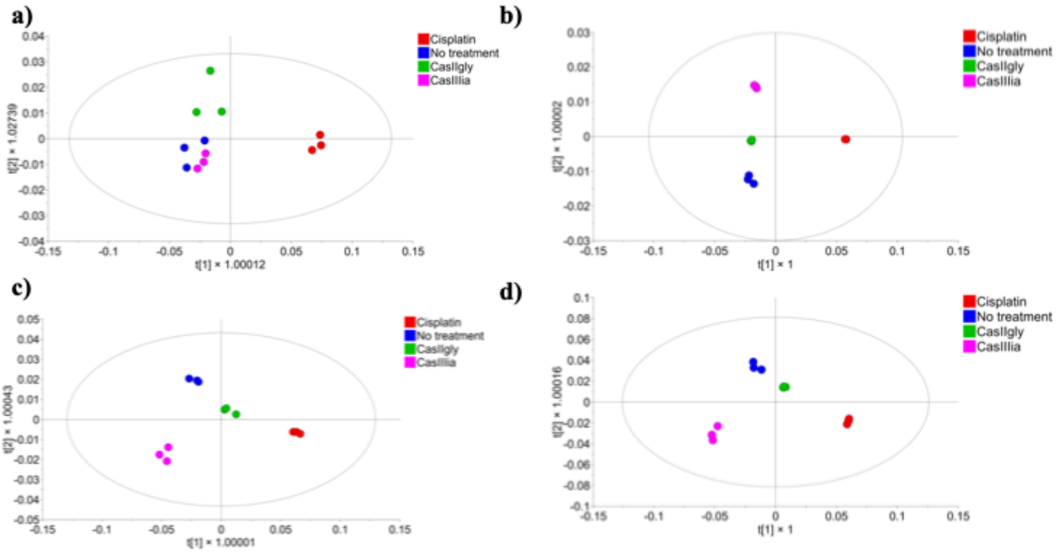

Figure S3. OPLS-DA score plots for samples collected at different treatment times: **(a)** 20 minutes treatment; **(b)** 1 hour; **(c)** 3 hours; **(d)** 6 hours.

Bucket (ppm)	Metabolites
3.22-3.20	Phosphocholine, Glycerophosphocholine, Acetylcholine
1.32-1.30	Lactate, Threonine
2.00-1.98	Acetamide
3.92-3.90	Creatine
3.04-3.02	Creatine, Phosphocreatine
2.36-2.34	Glutamate, Proline
1.34-1.32	Lactate, Threonine
2.08-2.06	Uridine diphosphate-N-acetylglucosamine, Acetylcysteine
4.74-4.72	NI
2.34-2.32	Glutamate, Proline, Glycylproline
4.02-4.00	Uridine diphosphate-N-acetylglucosamine
4.28-4.26	Glycylproline, Threonine, Uridine diphosphate-N-acetylglucosamine, NADH
3.20-3.18	Choline
1.54-1.52	NI

Table S3. Metabolites that contribute to the separation between untreated and cisplatin-treated samples at 20-minutes analysis.

Bucket (ppm)	Metabolites
1.32-1.30	Lactate, Threonine
3.22-3.20	Phosphocholine, Glycerophosphocholine
2.00-1.98	Acetamide
2.08-2.06	Glutamate, UDP-N-Acetylglucosamine
4.74-4.72	NI
8.46-8.44	Formate, NADH
3.04-3.02	Creatine, Phosphocreatine, Creatinine
3.86-3.84	Glycylproline, N-Acetylglucosamine
2.72-2.70	Dimethylamine

*NI: the metabolites corresponding to this bucket were not identified.

Table S4. Metabolites that contribute to the separation between untreated and CasIIIia-treated samples at 20 minutes analysis.

Bucket (ppm)	Metabolites
8.46-8.44	Formate, NADH
1.32-1.30	Lactate, Threonine
0.86-0.84	NI
2.36-2.34	Glutamate, Proline
0.88-0.86	NI
3.04-3.02	Creatine, Phosphocreatine, Creatinine
2.08-2.06	Glutamate, UDP-N-Acetylglucosamine, Acetylcysteine
2.34-2.32	Glutamate, Proline
3.60-3.58	Phosphocholine, NI
2.00-1.98	Acetamide
1.54-1.52	NI
2.40-2.38	Succinate, NI
3.94-3.92	Glycylproline, 1,7-Dimethylxantine

*NI: the metabolites corresponding to this bucket were not identified.

Table S5. Metabolites that contribute to the separation between untreated and CasIIgly-treated samples at 20-minutes analysis.

	Concentration mean (μM)	
**Metabolite**	**No treatment (20 min)**	**Cisplatin (20 min)**
Acetamide	0.0008 ± 0.0001	0.0016 ± 0.0001
Acetylcysteine	0.0003 ± 0.0001	0.0017 ± 0.0006
Acetylcholine	0.0040 ± 0.0001	0.0033 ± 0.0010
Choline	0.0012 ± 0.0002	0.0009 ± 0.0001
Creatine	0.0077 ± 0.0032	0.0023 ± 0.0006
Glutamate	0.0220 ± 0.0032	0.0123 ± 0.0030
Glycerophosphocholine	0.0011 ± 0.0001	0.0013 ± 0.0006
Glycylproline	0.0213 ± 0.0076	0.0180 ± 0.0010
Lactate	0.0193 ± 0.0035	0.0023 ± 0.0011
NADH	0.0050 ± 0.0017	0.0030 ± 0.0016
Phosphocreatine	0.0019 ± 0.0002	0.0018 ± 0.0001
Phosphocholine	0.0063 ± 0.0021	0.0027 ± 0.0006
Proline	0.0222 ± 0.0027	0.0210 ± 0.0070
Threonine	0.0203 ± 0.0037	0.0161 ± 0.0086
Uridine diphosphate-N-acetylglucosamine	0.0077 ± 0.0025	0.0030 ± 0.0010

Table S6. Concentration and standard deviation of metabolites that were found to be relevant in untreated and cisplatin-treated samples at 20-minutes analysis.

	Concentration mean (µM)	
**Metabolite**	**No treatment (20 min)**	**CasIIIa (20 min)**
Acetamide	0.0008 ± 0.0001	0.0012 ± 0.0004
Acetylcholine	0.0040 ± 0.0001	0.0043 ± 0.0015
Creatine	0.0077 ± 0.0032	0.0054 ± 0.0020
Creatinine	0.0010 ± 0.0001	0.0006 ± 0.0002
Dimethylamine	0.0011 ± 0.0004	0.0015 ± 0.0001
Formate	0.0017 ± 0.0006	0.0009 ± 0.0003
Glutamate	0.0220 ± 0.0032	0.0165 ± 0.0035
Glycerophosphocholine	0.0011 ± 0.0001	0.0019 ± 0.0004
Glycylproline	0.0213 ± 0.0076	0.0185 ± 0.0010
Lactate	0.0193 ± 0.0035	0.0039 ± 0.0010
NADH	0.0050 ± 0.0017	0.0027 ± 0.0010
N-Acetylglucosamine	0.0056 ± 0.0003	0.0034 ± 0.0007
Phosphocholine	0.0063 ± 0.0021	0.0050 ± 0.0017
Phosphocreatine	0.0019 ± 0.0010	0.0010 ± 0.0002
Threonine	0.0203 ± 0.0037	0.0123 ± 0.0022
UDP-N-Acetylglucosamine	0.0077 ± 0.0025	0.0049 ± 0.0016

Table S7. Concentration and standard deviation of metabolites that were found to be relevant in untreated and CasIIIia-treated samples at 20-minutes analysis.

	Concentration mean (μM)	
**Metabolite**	**No treatment (20 min)**	**CasIIgly (20 min)**
1,7-Dimethylxantine	0.0008 ± 0.0002	0.0015 ± 0.0003
Acetamide	0.0008 ± 0.0001	0.0016 ± 0.0002
Acetylcysteine	0.0003 ± 0.0001	0.0028 ± 0.0011
Creatine	0.0077 ± 0.0032	0.0044 ± 0.0028
Creatinine	0.0010 ± 0.0001	0.0008 ± 0.0002
Formate	0.0017 ± 0.0006	0.0013 ± 0.0006
Glutamate	0.0220 ± 0.0032	0.0136 ± 0.0035
Glycylproline	0.0213 ± 0.0076	0.0217 ± 0.0038
Lactate	0.0193 ± 0.0035	0.0037 ± 0.0014
NADH	0.0050 ± 0.0017	0.0033 ± 0.0025
Phosphocholine	0.0063 ± 0.0021	0.0052 ± 0.0011
Phosphocreatine	0.0019 ± 0.0010	0.0011 ± 0.0001
Proline	0.0222 ± 0.0027	0.0074 ± 0.0005
Succinate	0.0005 ± 0.0001	0.0009 ± 0.0003
Threonine	0.0203 ± 0.0037	0.0135 ± 0.0095
UDP-N-Acetylglucosamine	0.0077 ± 0.0025	0.0056 ± 0.0012

Table S8. Concentration and standard deviation of metabolites that were found to be relevant in untreated and CasIIgly-treated samples at 20-minutes analysis.

Bucket (ppm)	Metabolites
3.22-3.20	Phosphocholine, Glycerophosphocholine, Acetylcholine
2.36-2.34	Glutamate, Proline
2.34-2.32	Glutamate, Proline, Glycylproline
1.32-1.30	Lactate, Threonine
2.12-2.10	Glutamine, Glutamate, Glycylproline
2.06-2.04	Glutamate, Proline, Uridine diphosphate-N-acetylglucosamine
8.46-8.44	NADH, Formate
3.04-3.02	Tyrosine, Creatine, Creatinine, Phosphocreatine
2.14-2.12	Glutamine, Glutamate, Glycylproline
3.94-3.92	Glycylproline
2.04-2.02	Glutamate, Proline
3.60-3.58	Glycylproline, Threonine
4.16-4.14	Phosphocholine, N-Acetylglutamine
3.92-3.90	Glycylproline, Creatine
2.08-2.06	Proline, Uridine diphosphate-N-acetylglucosamine
3.42-3.40	Taurine
3.26-3.24	Arginine, Taurine

Table S9. Metabolites that contribute to the separation untreated samples from 20-minutes to 6-hours.

Bucket (ppm)	Metabolites
3.22-3.20	Phosphocholine, Glycerophosphocholine
1.32-1.30	Lactate, Threonine
2.36-2.34	Glutamate, Proline
8.46-8.44	NADH, Formate
1.48-1.46	Alanine
2.34-2.32	Glutamate, Proline, Glycylproline
2.08-2.06	Uridine diphosphate-N-acetylglucosamine, Acetylcysteine
3.04-3.02	Creatine, Phosphocreatine, Creatinine
3.26-3.24	Arginine, Taurine
1.34-1.32	Lactate, Threonine
2.12-2.10	Glutamate, Glycylproline, Glutamine, N-Acetylglutamine
3.60-3.58	Threonine, NI
4.28-4.26	Glycylproline, Threonine, Uridine diphosphate-N-acetylglucosamine, NADH
5.98-5.96	Uridine diphosphate-N-acetylglucosamine
3.42-3.40	Taurine
4.24-4.22	Uridine diphosphate-N-acetylglucosamine
4.16-4.14	Phosphocholine, N-Acetylglutamine
2.14-2.12	Glutamate, Glycylproline
3.82-3.80	Uridine diphosphate-N-acetylglucosamine
2.06-2.04	Glutamate, Proline
0.96-0.94	Leucine, Valine
3.94-3.92	Glycylproline, Phosphocreatine, Glycolate

*NI: the metabolites corresponding to this bucket were not identified.

Table S10. Metabolites that contribute to the separation cisplatin-treated samples from 20-minutes to 6-hours.

Bucket (ppm)	Metabolites
3.22-3.20	Phosphocholine, Glycerophosphocholine, Acetylcholine
1.32-1.30	Lactate, Threonine, NI
2.36-2.34	Glutamate, Proline, Glycylproline
2.08-2.06	Proline, Uridine diphosphate-N-acetylglucosamine
2.34-2.32	Proline, Glycylproline
3.04-3.02	Tyrosine, Creatine, Phosphocreatine, Creatinine
1.34-1.32	Lactate, Threonine, NI
2.12-2.10	Glutamate, Glycylproline, Glutamine, N-Acetylglutamine
3.26-3.24	Glucose, Arginine, Taurine
0.86-0.84	NI
2.14-2.12	Glycylproline, Glutamate, Glutamine
0.88-0.86	NI
3.60-3.58	Valine, Threonine, Glycylproline
2.06-2.04	Proline, Glutamate, Uridine diphosphate-N-acetylglucosamine
4.16-4.14	Phosphocholine, N-Acetylglutamine
5.98-5.96	Cytosine, Uridine diphosphate-N-acetylglucosamine
3.42-3.40	Glucose, Taurine

*NI: the metabolites corresponding to this bucket were not identified.

Table S11. Metabolites that contribute to the separation CasIIIia-treated samples from 20-minutes to 6-hours.

Bucket (ppm)	Metabolites
3.22-3.20	Phosphocholine, Glycerophosphocholine, Acetylcholine
1.32-1.30	Lactate, Threonine, NI
2.36-2.34	Glutamate, Proline, Glycylproline
1.34-1.32	Threonine, Lactate
2.34-2.32	Proline, Glycylproline
3.04-3.02	Tyrosine, Creatine, Phosphocreatine, Creatinine
2.08-2.06	Proline, Uridine diphosphate-N-acetylglucosamine
2.12-2.10	Glutamate, Glutamine, Glycylproline, NI
3.70-3.68	Glucose, NI
1.48-1.46	Alanine
2.06-2.04	Glutamate, Proline
3.92-3.90	Creatine, NI
2.00-1.98	Acetamide
2.14-2.12	Glycylproline, Glutamate
3.26-3.24	Arginine, Taurine
2.72-2.70	Dimethylamine
5.98-5.96	Uridine diphosphate-N-acetylglucosamine
4.16-4.14	N-Acetylglutamine, Phosphocholine

*NI: the metabolites corresponding to this bucket were not identified.

Table S12. Metabolites that contribute to the separation CasIIgly-treated samples from 20-minutes to 6-hours.

	Concentration mean (μM)	
**Metabolite**	**No treatment (20 min)**	**No treatment (6 h)**
Arginine	0.0067 ± 0.0015	0.0295 ± 0.0007
Creatine	0.0077 ± 0.0032	0.0075 ± 0.0007
Creatinine	0.0010 ± 0.0001	0.0017 ± 0.0002
Formate	0.0017 ± 0.0006	0.0015 ± 0.0007
Glutamate	0.0220 ± 0.0032	0.0130 ± 0.0028
Glutamine	0.0068 ± 0.0006	0.0065 ± 0.0007
Glycerophosphocholine	0.0011 ± 0.0001	0.0030 ± 0.0001
Glycylproline	0.0213 ± 0.0076	0.0210 ± 0.0002
Lactate	0.0193 ± 0.0035	0.0045 ± 0.0007
N-Acetylglutamine	0.0040 ± 0.0036	0.0073 ± 0.0004
NADH	0.0050 ± 0.0017	0.0065 ± 0.0007
Phosphocholine	0.0063 ± 0.0021	0.0145 ± 0.0035
Phosphocreatine	0.0019 ± 0.0002	0.0010 ± 0.0001
Proline	0.0222 ± 0.0027	0.0150 ± 0.0028
Taurine	0.0023 ± 0.0006	0.0050 ± 0.0004
Threonine	0.0203 ± 0.0037	0.0170 ± 0.0014
Tyrosine	0.0066 ± 0.0006	0.0065 ± 0.0003
Uridine diphosphate-N-acetylglucosamine	0.0077 ± 0.0025	0.0125 ± 0.0021

Table S13. Concentration and standard deviation of metabolites that were found to be relevant in 20-minutes to 6 hours analysis in untreated samples.

	Concentration mean (µM)	
**Metabolite**	**Cisplatin (20 min)**	**Cisplatin (6 h)**
Acetylcysteine	0.0017 ± 0.0006	0.0013 ± 0.0006
Alanine	0.0024 ± 0.0001	0.0023 ± 0.0001
Arginine	0.0217 ± 0.0025	0.0210 ± 0.0003
Creatine	0.0023 ± 0.0006	0.0000 ± 0.0000
Creatinine	0.0010 ± 0.0000	0.0007 ± 0.0001
Formate	0.0010 ± 0.0000	0.0020 ± 0.0001
Glutamate	0.0123 ± 0.0030	0.0088 ± 0.0009
Glutamine	0.0042 ± 0.0016	0.0050 ± 0.0006
Glycerophosphocholine	0.0013 ± 0.0006	0.0020 ± 0.0000
Glycolate	0.0014 ± 0.0001	0.0012 ± 0.0003
Glycylproline	0.0180 ± 0.0010	0.0157 ± 0.0031
Lactate	0.0023 ± 0.0011	0.0028 ± 0.0006
Leucine	0.0061 ± 0.0019	0.0064 ± 0.0006
N-Acetylglutamine	0.0030 ± 0.0010	0.0033 ± 0.0006
NADH	0.0030 ± 0.0016	0.0026 ± 0.0003
Phosphocreatine	0.0018 ± 0.0001	0.0010 ± 0.0000
Phosphocholine	0.0027 ± 0.0006	0.0040 ± 0.0010
Proline	0.0210 ± 0.0070	0.0109 ± 0.0017
Taurine	0.0067 ± 0.0015	0.0033 ± 0.0015
Threonine	0.0161 ± 0.0086	0.0143 ± 0.0019
Uridine diphosphate-N-acetylglucosamine	0.0030 ± 0.0010	0.0047 ± 0.0006
Valine	0.0025 ± 0.0008	0.0039 ± 0.0013

Table S14. Concentration and standard deviation of metabolites that were found to be relevant in 20-minutes to 6 hours analysis in cisplatin-treated samples.

Metabolite	Concentration mean (μM)	
	CasIIIia (20 min)	CasIIIia (6 h)
Acetylcholine	0.0043 ± 0.0015	0.0048 ± 0.0003
Arginine	0.0148 ± 0.0019	0.0100 ± 0.0005
Creatine	0.0054 ± 0.0020	0.0025 ± 0.0008
Creatinine	0.0006 ± 0.0002	0.0004 ± 0.0001
Cytosine	0.0004 ± 0.0001	0.0007 ± 0.0003
Glutamate	0.0165 ± 0.0035	0.0080 ± 0.0019
Glutamine	0.0048 ± 0.0006	0.0030 ± 0.0011
Glycerophosphocholine	0.0019 ± 0.0004	0.0031 ± 0.0007
Glycylproline	0.0185 ± 0.0010	0.0136 ± 0.0021
Lactate	0.0039 ± 0.0010	0.0037 ± 0.0007
N-Acetylglutamine	0.0034 ± 0.0007	0.0027 ± 0.0005
Phosphocholine	0.0050 ± 0.0017	0.0059 ± 0.0023
Phosphocreatine	0.0010 ± 0.0002	0.0005 ± 0.0002
Proline	0.0141 ± 0.0040	0.0075 ± 0.0008
Taurine	0.0045 ± 0.0013	0.0021 ± 0.0004
Threonine	0.0123 ± 0.0022	0.0096 ± 0.0002
Tyrosine	0.0034 ± 0.0022	0.0022 ± 0.0008
UDP-N-Acetylglucosamine	0.0049 ± 0.0016	0.0062 ± 0.0018
Valine	0.0029 ± 0.0012	0.0026 ± 0.0012

Table S15. Concentration and standard deviation of metabolites that were found to be relevant in 20-minutes to 6 hours analysis in CasIIIia-treated samples.

	Concentration mean (μM)	
**Metabolite**	**CasIIgly (20 min)**	**CasIIgly (6 h)**
1,7-Dimethylxantine	0.0015 ± 0.0003	0.0013 ± 0.0004
Acetamide	0.0016 ± 0.0002	0.0013 ± 0.0005
Acetylcysteine	0.0028 ± 0.0011	0.0017 ± 0.0005
Acetylcholine	0.0048 ± 0.0003	0.0034 ± 0.0003
Alanine	0.0027 ± 0.0001	0.0021 ± 0.0002
Arginine	0.0275 ± 0.0031	0.0185 ± 0.0006
Creatine	0.0044 ± 0.0028	0.0026 ± 0.0008
Creatinine	0.0008 ± 0.0002	0.0000 ± 0.0000
Dimethylamine	0.0030 ± 0.0003	0.0027 ± 0.0005
Formate	0.0013 ± 0.0006	0.0022 ± 0.0009
Glucose	0.0097 ± 0.0059	0.0240 ± 0.0042
Glutamate	0.0136 ± 0.0035	0.0064 ± 0.0039
Glutamine	0.0054 ± 0.0016	0.0161 ± 0.0028
Glycerophosphocholine	0.0022 ± 0.0007	0.0016 ± 0.0009
Glycylproline	0.0217 ± 0.0038	0.0144 ± 0.0019
Lactate	0.0037 ± 0.0014	0.0146 ± 0.0023
Leucine	0.0043 ± 0.0006	0.0044 ± 0.0026
Lysine	0.0074 ± 0.0055	0.0054 ± 0.0030
NADH	0.0033 ± 0.0025	0.0030 ± 0.0001
N-Acetylglutamine	0.0037 ± 0.0010	0.0021 ± 0.0002
Phosphocreatine	0.0011 ± 0.0001	0.0007 ± 0.0004
Phosphocholine	0.0052 ± 0.0011	0.0042 ± 0.0028
Proline	0.0074 ± 0.0005	0.0128 ± 0.0025
Succinate	0.0009 ± 0.0003	0.0005 ± 0.0001
Taurine	0.0062 ± 0.0020	0.0024 ± 0.0004
Threonine	0.0135 ± 0.0095	0.0154 ± 0.0070
Tyrosine	0.0039 ± 0.0011	0.0037 ± 0.0002
Uridine diphosphate-N-acetylglucosamine	0.0056 ± 0.0012	0.0050 ± 0.0018

Table S16. Concentration and standard deviation of metabolites that were found to be relevant in 20-minutes to 6 hours analysis in CasIIgly-treated samples.

	Total Cmpd	Hits	Statistic Q	Expected Q	Raw p	Holm p	FDR
Warburg Effect	57	3	47.234	20	0.024602	1	0.11793
Pyruvate Metabolism	47	2	45.575	20	0.04879	1	0.11793
Gluconeogenesis	33	2	45.575	20	0.04879	1	0.11793
Tyrosine Metabolism	70	2	51.886	20	0.072582	1	0.11793
Beta-Alanine Metabolism	34	2	51.886	20	0.072582	1	0.11793
Ammonia Recycling	31	2	51.886	20	0.072582	1	0.11793
Cysteine Metabolism	26	2	51.886	20	0.072582	1	0.11793
Propanoate Metabolism	42	2	51.886	20	0.072582	1	0.11793
Valine, Leucine and Isoleucine Degradation	59	2	51.886	20	0.072582	1	0.11793
Lysine Degradation	30	2	51.886	20	0.072582	1	0.11793
Histidine Metabolism	42	2	51.886	20	0.072582	1	0.11793
Nicotinate and Nicotinamide Metabolism	35	2	51.886	20	0.072582	1	0.11793
Purine Metabolism	73	2	51.886	20	0.072582	1	0.11793
Folate Metabolism	29	2	51.886	20	0.072582	1	0.11793
Urea Cycle	28	2	51.886	20	0.072582	1	0.11793
Tryptophan Metabolism	59	2	51.886	20	0.072582	1	0.11793
Glutamate Metabolism	48	2	51.886	20	0.072582	1	0.11793
Glucose-Alanine Cycle	13	2	51.886	20	0.072582	1	0.11793
Malate-Aspartate Shuttle	10	2	51.886	20	0.072582	1	0.11793
Arginine and Proline Metabolism	52	4	48.864	20	0.085093	1	0.11793
Phenylalanine and Tyrosine Metabolism	27	1	55.449	20	0.089487	1	0.11793
Glutathione Metabolism	20	1	55.449	20	0.089487	1	0.11793
Alanine Metabolism	17	1	55.449	20	0.089487	1	0.11793
Aspartate Metabolism	35	1	55.449	20	0.089487	1	0.11793
Arachidonic Acid Metabolism	67	1	55.449	20	0.089487	1	0.11793
Glycine and Serine Metabolism	59	4	45.165	20	0.092766	1	0.11793
Pterine Biosynthesis	28	1	50	20	0.11612	1	0.11793
Nucleotide Sugars Metabolism	20	1	50	20	0.11612	1	0.11793
Porphyrin Metabolism	40	1	50	20	0.11612	1	0.11793
Phospholipid Biosynthesis	29	2	50	20	0.11612	1	0.11793
Caffeine Metabolism	23	1	50	20	0.11612	1	0.11793
Methionine Metabolism	42	1	50	20	0.11612	1	0.11793
Sphingolipid Metabolism	40	1	50	20	0.11612	1	0.11793
Bile Acid Biosynthesis	65	1	50	20	0.11612	1	0.11793
Glycerolipid Metabolism	25	1	50	20	0.11612	1	0.11793
Glycolysis	23	1	50	20	0.11612	1	0.11793

Galactose Metabolism	38	1	50	20	0.11612	1	0.11793
Fatty acid Metabolism	43	1	50	20	0.11612	1	0.11793
Fatty Acid Elongation In Mitochondria	35	1	50	20	0.11612	1	0.11793
Citric Acid Cycle	32	1	50	20	0.11612	1	0.11793
Starch and Sucrose Metabolism	31	1	50	20	0.11612	1	0.11793
Fructose and Mannose Degradation	31	1	50	20	0.11612	1	0.11793
Androgen and Estrogen Metabolism	33	1	50	20	0.11612	1	0.11793
Ketone Body Metabolism	13	1	50	20	0.11612	1	0.11793
Butyrate Metabolism	19	1	50	20	0.11612	1	0.11793
Retinol Metabolism	35	1	50	20	0.11612	1	0.11793
Betaine Metabolism	21	1	50	20	0.11612	1	0.11793
Glycerol Phosphate Shuttle	11	1	50	20	0.11612	1	0.11793
Steroidogenesis	43	1	50	20	0.11612	1	0.11793
Mitochondrial Electron Transport Chain	19	1	50	20	0.11612	1	0.11793
Ethanol Degradation	19	1	50	20	0.11612	1	0.11793
Phytanic Acid Peroxisomal Oxidation	26	1	50	20	0.11612	1	0.11793
Carnitine Synthesis	22	1	50	20	0.11612	1	0.11793
Transfer of Acetyl Groups into Mitochondria	22	1	50	20	0.11612	1	0.11793
Plasmalogen Synthesis	26	1	50	20	0.11612	1	0.11793
Mitochondrial Beta-Oxidation of Short Chain Saturated Fatty Acids	27	1	50	20	0.11612	1	0.11793
Mitochondrial Beta-Oxidation of Medium Chain Saturated Fatty Acids	27	1	50	20	0.11612	1	0.11793
Mitochondrial Beta-Oxidation of Long Chain Saturated Fatty Acids	28	1	50	20	0.11612	1	0.11793
Phosphatidylcholine Biosynthesis	14	1	50	20	0.11612	1	0.11793
De Novo Triacylglycerol Biosynthesis	9	1	50	20	0.11612	1	0.11793
Cardiolipin Biosynthesis	11	1	50	20	0.11612	1	0.11793
Androstenedione Metabolism	24	1	50	20	0.11612	1	0.11793
Estrone Metabolism	24	1	50	20	0.11612	1	0.11793
Threonine and 2-Oxobutanoate Degradation	20	2	38.445	20	0.12953	1	0.12953

Table S17. Enrichment of metabolite sets without treatment vs. cisplatin treatment at 20 minutes.

	Total Cmpd	Hits	Statistic Q	Expected Q	Raw p	Holm p	FDR
Amino Sugar Metabolism	33	1	90.21	20	0.0037186	0.19337	0.064456
Pyruvate Metabolism	47	2	61.575	20	0.0063399	0.31065	0.082418
Gluconeogenesis	33	3	60.747	20	0.029873	1	0.22192
Warburg Effect	57	3	60.747	20	0.029873	1	0.22192
Retinol Metabolism	35	2	9.6701	20	0.70228	1	0.81114
Pterine Biosynthesis	28	2	8.5534	20	0.71774	1	0.81114
Folate Metabolism	29	2	8.5534	20	0.71774	1	0.81114
Tryptophan Metabolism	59	2	8.5534	20	0.71774	1	0.81114
Androgen and Estrogen Metabolism	33	2	8.5534	20	0.71774	1	0.81114
Androstenedione Metabolism	24	2	8.5534	20	0.71774	1	0.81114
Arginine and Proline Metabolism	52	3	8.998	20	0.74731	1	0.81114
Phospholipid Biosynthesis	29	3	8.1583	20	0.74893	1	0.81114
Tyrosine Metabolism	70	1	2.6506	20	0.75795	1	0.81114
Beta-Alanine Metabolism	34	1	2.6506	20	0.75795	1	0.81114
Ammonia Recycling	31	1	2.6506	20	0.75795	1	0.81114
Nucleotide Sugars Metabolism	20	1	2.6506	20	0.75795	1	0.81114
Cysteine Metabolism	26	1	2.6506	20	0.75795	1	0.81114
Propanoate Metabolism	42	1	2.6506	20	0.75795	1	0.81114
Porphyrin Metabolism	40	1	2.6506	20	0.75795	1	0.81114
Caffeine Metabolism	23	1	2.6506	20	0.75795	1	0.81114
Valine, Leucine and Isoleucine Degradation	59	1	2.6506	20	0.75795	1	0.81114
Methionine Metabolism	42	1	2.6506	20	0.75795	1	0.81114
Bile Acid Biosynthesis	65	1	2.6506	20	0.75795	1	0.81114
Lysine Degradation	30	1	2.6506	20	0.75795	1	0.81114
Glycerolipid Metabolism	25	1	2.6506	20	0.75795	1	0.81114
Glycolysis	23	1	2.6506	20	0.75795	1	0.81114
Galactose Metabolism	38	1	2.6506	20	0.75795	1	0.81114
Histidine Metabolism	42	1	2.6506	20	0.75795	1	0.81114
Nicotinate and Nicotinamide Metabolism	35	1	2.6506	20	0.75795	1	0.81114
Purine Metabolism	73	1	2.6506	20	0.75795	1	0.81114
Fatty acid Metabolism	43	1	2.6506	20	0.75795	1	0.81114
Fatty Acid Elongation In Mitochondria	35	1	2.6506	20	0.75795	1	0.81114
Citric Acid Cycle	32	1	2.6506	20	0.75795	1	0.81114

Starch and Sucrose Metabolism	31	1	2.6506	20	0.75795	1	0.81114
Urea Cycle	28	1	2.6506	20	0.75795	1	0.81114
Fructose and Mannose Degradation	31	1	2.6506	20	0.75795	1	0.81114
Ketone Body Metabolism	13	1	2.6506	20	0.75795	1	0.81114
Glutamate Metabolism	48	1	2.6506	20	0.75795	1	0.81114
Butyrate Metabolism	19	1	2.6506	20	0.75795	1	0.81114
Betaine Metabolism	21	1	2.6506	20	0.75795	1	0.81114
Glycerol Phosphate Shuttle	11	1	2.6506	20	0.75795	1	0.81114
Glucose-Alanine Cycle	13	1	2.6506	20	0.75795	1	0.81114
Malate-Aspartate Shuttle	10	1	2.6506	20	0.75795	1	0.81114
Steroidogenesis	43	1	2.6506	20	0.75795	1	0.81114
Mitochondrial Electron Transport Chain	19	1	2.6506	20	0.75795	1	0.81114
Ethanol Degradation	19	1	2.6506	20	0.75795	1	0.81114
Phytanic Acid Peroxisomal Oxidation	26	1	2.6506	20	0.75795	1	0.81114
Carnitine Synthesis	22	1	2.6506	20	0.75795	1	0.81114
Transfer of Acetyl Groups into Mitochondria	22	1	2.6506	20	0.75795	1	0.81114
Plasmalogen Synthesis	26	1	2.6506	20	0.75795	1	0.81114
Mitochondrial Beta-Oxidation of Short Chain Saturated Fatty Acids	27	1	2.6506	20	0.75795	1	0.81114
Mitochondrial Beta-Oxidation of Medium Chain Saturated Fatty Acids	27	1	2.6506	20	0.75795	1	0.81114
Mitochondrial Beta-Oxidation of Long Chain Saturated Fatty Acids	28	1	2.6506	20	0.75795	1	0.81114
De Novo Triacylglycerol Biosynthesis	9	1	2.6506	20	0.75795	1	0.81114
Cardiolipin Biosynthesis	11	1	2.6506	20	0.75795	1	0.81114
Estrone Metabolism	24	1	2.6506	20	0.75795	1	0.81114
Glycine and Serine Metabolism	59	3	3.3753	20	0.94621	1	0.96228
Threonine and 2-Oxobutanoate Degradation	20	2	0.79145	20	0.95033	1	0.96228
Sphingolipid Metabolism	40	1	0.063275	20	0.96228	1	0.96228
Phosphatidylcholine Biosynthesis	14	1	0.063275	20	0.96228	1	0.96228

Table S18. Enrichment of metabolite sets without treatment vs. CasIIIia treatment at 20 minutes.

	Total Cmpd	Hits	Statistic Q	Expected Q	Raw p	Holm p	FDR
Amino Sugar Metabolism	33	1	73.517	20	0.029044	1	0.19847
Steroid Biosynthesis	48	1	73.517	20	0.029044	1	0.19847
Pyruvate Metabolism	47	1	73.517	20	0.029044	1	0.19847
Gluconeogenesis	33	1	73.517	20	0.029044	1	0.19847
Warburg Effect	57	1	73.517	20	0.029044	1	0.19847
Pterine Biosynthesis	28	1	73.517	20	0.029044	1	0.19847
Folate Metabolism	29	3	65.164	20	0.034178	1	0.20018
Tryptophan Metabolism	59	2	9.9	20	0.66679	1	0.81825
Androgen and Estrogen Metabolism	33	2	9.9	20	0.66679	1	0.81825
Androstenedione Metabolism	24	2	9.9	20	0.66679	1	0.81825
Arginine and Proline Metabolism	52	4	12.098	20	0.69506	1	0.81825
Tyrosine Metabolism	70	1	2.8037	20	0.75118	1	0.81825
Beta-Alanine Metabolism	34	1	2.8037	20	0.75118	1	0.81825
Ammonia Recycling	31	1	2.8037	20	0.75118	1	0.81825
Nucleotide Sugars Metabolism	20	1	2.8037	20	0.75118	1	0.81825
Cysteine Metabolism	26	1	2.8037	20	0.75118	1	0.81825
Propanoate Metabolism	42	1	2.8037	20	0.75118	1	0.81825
Porphyrin Metabolism	40	1	2.8037	20	0.75118	1	0.81825
Caffeine Metabolism	23	1	2.8037	20	0.75118	1	0.81825
Valine, Leucine and Isoleucine Degradation	59	1	2.8037	20	0.75118	1	0.81825
Methionine Metabolism	42	1	2.8037	20	0.75118	1	0.81825
Bile Acid Biosynthesis	65	1	2.8037	20	0.75118	1	0.81825
Lysine Degradation	30	1	2.8037	20	0.75118	1	0.81825
Glycerolipid Metabolism	25	1	2.8037	20	0.75118	1	0.81825
Glycolysis	23	1	2.8037	20	0.75118	1	0.81825
Galactose Metabolism	38	1	2.8037	20	0.75118	1	0.81825
Histidine Metabolism	42	1	2.8037	20	0.75118	1	0.81825
Nicotinate and Nicotinamide Metabolism	35	1	2.8037	20	0.75118	1	0.81825
Purine Metabolism	73	1	2.8037	20	0.75118	1	0.81825
Fatty acid Metabolism	43	1	2.8037	20	0.75118	1	0.81825
Fatty Acid Elongation In Mitochondria	35	1	2.8037	20	0.75118	1	0.81825

Citric Acid Cycle	32	1	2.8037	20	0.75118	1	0.81825
Starch and Sucrose Metabolism	31	1	2.8037	20	0.75118	1	0.81825
Urea Cycle	28	1	2.8037	20	0.75118	1	0.81825
Fructose and Mannose Degradation	31	1	2.8037	20	0.75118	1	0.81825
Ketone Body Metabolism	13	1	2.8037	20	0.75118	1	0.81825
Glutamate Metabolism	48	1	2.8037	20	0.75118	1	0.81825
Butyrate Metabolism	19	1	2.8037	20	0.75118	1	0.81825
Retinol Metabolism	35	1	2.8037	20	0.75118	1	0.81825
Betaine Metabolism	21	1	2.8037	20	0.75118	1	0.81825
Glycerol Phosphate Shuttle	11	1	2.8037	20	0.75118	1	0.81825
Glucose-Alanine Cycle	13	1	2.8037	20	0.75118	1	0.81825
Malate-Aspartate Shuttle	10	1	2.8037	20	0.75118	1	0.81825
Steroidogenesis	43	1	2.8037	20	0.75118	1	0.81825
Mitochondrial Electron Transport Chain	19	1	2.8037	20	0.75118	1	0.81825
Ethanol Degradation	19	1	2.8037	20	0.75118	1	0.81825
Phytanic Acid Peroxisomal Oxidation	26	1	2.8037	20	0.75118	1	0.81825
Carnitine Synthesis	22	1	2.8037	20	0.75118	1	0.81825
Transfer of Acetyl Groups into Mitochondria	22	1	2.8037	20	0.75118	1	0.81825
Plasmalogen Synthesis	26	1	2.8037	20	0.75118	1	0.81825
Mitochondrial Beta-Oxidation of Short Chain Saturated Fatty Acids	27	1	2.8037	20	0.75118	1	0.81825
Mitochondrial Beta-Oxidation of Medium Chain Saturated Fatty Acids	27	1	2.8037	20	0.75118	1	0.81825
Mitochondrial Beta-Oxidation of Long Chain Saturated Fatty Acids	28	1	2.8037	20	0.75118	1	0.81825
De Novo Triacylglycerol Biosynthesis	9	1	2.8037	20	0.75118	1	0.81825
Cardiolipin Biosynthesis	11	1	2.8037	20	0.75118	1	0.81825
Estrone Metabolism	24	1	2.8037	20	0.75118	1	0.81825
Sphingolipid Metabolism	40	1	0.5884	20	0.88517	1	0.93095
Phosphatidylcholine Biosynthesis	14	1	0.5884	20	0.88517	1	0.93095
Glycine and Serine Metabolism	59	3	5.8377	20	0.91485	1	0.94533

Threonine and 2-Oxobutanoate Degradation	20	2	1.8597	20	0.93015	1	0.94533
Phospholipid Biosynthesis	29	2	1.8047	20	0.94533	1	0.94533

Table S19. Enrichment of metabolite sets without treatment vs. CasIIgly treatment at 20 minutes.

	Total Cmpd	Hits	Statistic Q	Expected Q	Raw p	Holm p	FDR
Sphingolipid Metabolism	40	1	61.72	25	0.11525	1	0.7442
Phosphatidylcholine Biosynthesis	14	1	61.72	25	0.11525	1	0.7442
Phospholipid Biosynthesis	29	2	45.636	25	0.19765	1	0.7442
Steroid Biosynthesis	48	1	42.782	25	0.23114	1	0.7442
Pterine Biosynthesis	28	2	31.727	25	0.30761	1	0.7442
Androgen and Estrogen Metabolism	33	2	31.727	25	0.30761	1	0.7442
Androstenedione Metabolism	24	2	31.727	25	0.30761	1	0.7442
Catecholamine Biosynthesis	20	1	23.514	25	0.40772	1	0.7442
Thyroid hormone synthesis	13	1	23.514	25	0.40772	1	0.7442
Amino Sugar Metabolism	33	3	24.799	25	0.4143	1	0.7442
Folate Metabolism	29	3	27.718	25	0.43129	1	0.7442
Tryptophan Metabolism	59	3	27.718	25	0.43129	1	0.7442
Nucleotide Sugars Metabolism	20	1	19.16	25	0.46102	1	0.7442
Porphyrin Metabolism	40	1	19.16	25	0.46102	1	0.7442
Caffeine Metabolism	23	1	19.16	25	0.46102	1	0.7442
Methionine Metabolism	42	1	19.16	25	0.46102	1	0.7442
Glycerolipid Metabolism	25	1	19.16	25	0.46102	1	0.7442
Glycolysis	23	1	19.16	25	0.46102	1	0.7442
Galactose Metabolism	38	1	19.16	25	0.46102	1	0.7442
Fatty acid Metabolism	43	1	19.16	25	0.46102	1	0.7442
Fatty Acid Elongation In Mitochondria	35	1	19.16	25	0.46102	1	0.7442
Citric Acid Cycle	32	1	19.16	25	0.46102	1	0.7442
Starch and Sucrose Metabolism	31	1	19.16	25	0.46102	1	0.7442
Fructose and Mannose Degradation	31	1	19.16	25	0.46102	1	0.7442
Ketone Body Metabolism	13	1	19.16	25	0.46102	1	0.7442
Butyrate Metabolism	19	1	19.16	25	0.46102	1	0.7442
Betaine Metabolism	21	1	19.16	25	0.46102	1	0.7442
Glycerol Phosphate Shuttle	11	1	19.16	25	0.46102	1	0.7442
Steroidogenesis	43	1	19.16	25	0.46102	1	0.7442
Mitochondrial Electron Transport Chain	19	1	19.16	25	0.46102	1	0.7442
Ethanol Degradation	19	1	19.16	25	0.46102	1	0.7442
Phytanic Acid Peroxisomal Oxidation	26	1	19.16	25	0.46102	1	0.7442
Carnitine Synthesis	22	1	19.16	25	0.46102	1	0.7442
Transfer of Acetyl Groups into Mitochondria	22	1	19.16	25	0.46102	1	0.7442
Plasmalogen Synthesis	26	1	19.16	25	0.46102	1	0.7442

Mitochondrial Beta-Oxidation of Short Chain Saturated Fatty Acids	27	1	19.16	25	0.46102	1	0.7442
Mitochondrial Beta-Oxidation of Medium Chain Saturated Fatty Acids	27	1	19.16	25	0.46102	1	0.7442
Mitochondrial Beta-Oxidation of Long Chain Saturated Fatty Acids	28	1	19.16	25	0.46102	1	0.7442
De Novo Triacylglycerol Biosynthesis	9	1	19.16	25	0.46102	1	0.7442
Cardiolipin Biosynthesis	11	1	19.16	25	0.46102	1	0.7442
Estrone Metabolism	24	1	19.16	25	0.46102	1	0.7442
Glutathione Metabolism	20	1	19.147	25	0.46119	1	0.7442
Alanine Metabolism	17	1	19.147	25	0.46119	1	0.7442
Arachidonic Acid Metabolism	67	1	19.147	25	0.46119	1	0.7442
Phenylalanine and Tyrosine Metabolism	27	2	21.196	25	0.54009	1	0.75299
Retinol Metabolism	35	2	13.851	25	0.54538	1	0.75299
Threonine and 2-Oxobutanoate Degradation	20	2	13.839	25	0.55335	1	0.75299
Beta-Alanine Metabolism	34	2	19.153	25	0.59649	1	0.75299
Cysteine Metabolism	26	2	19.153	25	0.59649	1	0.75299
Propanoate Metabolism	42	2	19.153	25	0.59649	1	0.75299
Valine, Leucine and Isoleucine Degradation	59	2	19.153	25	0.59649	1	0.75299
Lysine Degradation	30	2	19.153	25	0.59649	1	0.75299
Histidine Metabolism	42	2	19.153	25	0.59649	1	0.75299
Glucose-Alanine Cycle	13	2	19.153	25	0.59649	1	0.75299
Malate-Aspartate Shuttle	10	2	19.153	25	0.59649	1	0.75299
Tyrosine Metabolism	70	3	20.489	25	0.64889	1	0.75299
Arginine and Proline Metabolism	52	6	18.235	25	0.65451	1	0.75299
Pyruvate Metabolism	47	2	7.7037	25	0.65839	1	0.75299
Gluconeogenesis	33	2	7.7037	25	0.65839	1	0.75299
Ammonia Recycling	31	3	16.849	25	0.66815	1	0.75299
Nicotinate and Nicotinamide Metabolism	35	3	16.849	25	0.66815	1	0.75299
Purine Metabolism	73	3	16.849	25	0.66815	1	0.75299
Glutamate Metabolism	48	3	16.849	25	0.66815	1	0.75299
Pyrimidine Metabolism	57	1	5.0031	25	0.7176	1	0.7786
Phenylacetate Metabolism	9	1	5.0031	25	0.7176	1	0.7786
Urea Cycle	28	4	13.959	25	0.72377	1	0.7786
Warburg Effect	57	4	9.5542	25	0.75897	1	0.80428
Aspartate Metabolism	35	3	11.276	25	0.78251	1	0.81054
Glycine and Serine Metabolism	59	5	10.148	25	0.78771	1	0.81054

Bile Acid Biosynthesis	65	2	6.8466	25	0.8528	1	0.86498
Taurine and Hypotaurine Metabolism	12	1	0.0027054	25	0.99338	1	0.99338

Table S20. Enrichment of metabolite sets without treatment at 20 minutes and 6 hours.

	Total Cmpd	Hits	Statistic Q	Expected Q	Raw p	Holm p	FDR
Tyrosine Metabolism	70	2	61.835	20	0.035256	1	0.13889
Beta-Alanine Metabolism	34	2	61.835	20	0.035256	1	0.13889
Cysteine Metabolism	26	2	61.835	20	0.035256	1	0.13889
Lysine Degradation	30	2	61.835	20	0.035256	1	0.13889
Histidine Metabolism	42	2	61.835	20	0.035256	1	0.13889
Malate-Aspartate Shuttle	10	2	61.835	20	0.035256	1	0.13889
Propanoate Metabolism	42	3	53.856	20	0.039791	1	0.13889
Folate Metabolism	29	3	54.947	20	0.046048	1	0.13889
Ammonia Recycling	31	3	53.841	20	0.047628	1	0.13889
Nicotinate and Nicotinamide Metabolism	35	3	53.841	20	0.047628	1	0.13889
Purine Metabolism	73	3	53.841	20	0.047628	1	0.13889
Glucose-Alanine Cycle	13	3	55.058	20	0.054614	1	0.13889
Phenylalanine and Tyrosine Metabolism	27	1	64.286	20	0.055041	1	0.13889
Arachidonic Acid Metabolism	67	1	64.286	20	0.055041	1	0.13889
Warburg Effect	57	4	49.235	20	0.056323	1	0.13889
Tryptophan Metabolism	59	4	50.26	20	0.062105	1	0.13889
Valine, Leucine and Isoleucine Degradation	59	4	43.456	20	0.06868	1	0.13889
Glutamate Metabolism	48	4	49.776	20	0.069389	1	0.13889
Glutathione Metabolism	20	2	55.487	20	0.071352	1	0.13889
Alanine Metabolism	17	2	55.487	20	0.071352	1	0.13889
Amino Sugar Metabolism	33	3	41.839	20	0.078917	1	0.13889
Arginine and Proline Metabolism	52	5	43.651	20	0.092409	1	0.13889
Nucleotide Sugars Metabolism	20	1	52.941	20	0.10119	1	0.13889
Porphyrin Metabolism	40	1	52.941	20	0.10119	1	0.13889
Caffeine Metabolism	23	1	52.941	20	0.10119	1	0.13889
Methionine Metabolism	42	1	52.941	20	0.10119	1	0.13889
Glycerolipid Metabolism	25	1	52.941	20	0.10119	1	0.13889
Glycolysis	23	1	52.941	20	0.10119	1	0.13889
Galactose Metabolism	38	1	52.941	20	0.10119	1	0.13889
Fatty acid Metabolism	43	1	52.941	20	0.10119	1	0.13889

Fatty Acid Elongation In Mitochondria	35	1	52.941	20	0.10119	1	0.13889
Citric Acid Cycle	32	1	52.941	20	0.10119	1	0.13889
Starch and Sucrose Metabolism	31	1	52.941	20	0.10119	1	0.13889
Fructose and Mannose Degradation	31	1	52.941	20	0.10119	1	0.13889
Ketone Body Metabolism	13	1	52.941	20	0.10119	1	0.13889
Butyrate Metabolism	19	1	52.941	20	0.10119	1	0.13889
Betaine Metabolism	21	1	52.941	20	0.10119	1	0.13889
Glycerol Phosphate Shuttle	11	1	52.941	20	0.10119	1	0.13889
Steroidogenesis	43	1	52.941	20	0.10119	1	0.13889
Mitochondrial Electron Transport Chain	19	1	52.941	20	0.10119	1	0.13889
Ethanol Degradation	19	1	52.941	20	0.10119	1	0.13889
Phytanic Acid Peroxisomal Oxidation	26	1	52.941	20	0.10119	1	0.13889
Carnitine Synthesis	22	1	52.941	20	0.10119	1	0.13889
Transfer of Acetyl Groups into Mitochondria	22	1	52.941	20	0.10119	1	0.13889
Plasmalogen Synthesis	26	1	52.941	20	0.10119	1	0.13889
Mitochondrial Beta-Oxidation of Short Chain Saturated Fatty Acids	27	1	52.941	20	0.10119	1	0.13889
Mitochondrial Beta-Oxidation of Medium Chain Saturated Fatty Acids	27	1	52.941	20	0.10119	1	0.13889
Mitochondrial Beta-Oxidation of Long Chain Saturated Fatty Acids	28	1	52.941	20	0.10119	1	0.13889
De Novo Triacylglycerol Biosynthesis	9	1	52.941	20	0.10119	1	0.13889
Cardiolipin Biosynthesis	11	1	52.941	20	0.10119	1	0.13889
Estrone Metabolism	24	1	52.941	20	0.10119	1	0.13889
Urea Cycle	28	5	34.241	20	0.14416	1	0.19202
Glycine and Serine Metabolism	59	6	35.788	20	0.14982	1	0.19202
Pterine Biosynthesis	28	2	35.324	20	0.15362	1	0.19202
Androgen and Estrogen Metabolism	33	2	35.324	20	0.15362	1	0.19202
Androstenedione Metabolism	24	2	35.324	20	0.15362	1	0.19202
Threonine and 2-Oxobutanoate Degradation	20	2	41.349	20	0.16166	1	0.19852
Aspartate Metabolism	35	3	32.502	20	0.1801	1	0.21736
Pyruvate Metabolism	47	2	35.324	20	0.19127	1	0.22315
Gluconeogenesis	33	2	35.324	20	0.19127	1	0.22315
Bile Acid Biosynthesis	65	2	30.489	20	0.2259	1	0.25607
Retinol Metabolism	35	2	31.401	20	0.22681	1	0.25607
Phospholipid Biosynthesis	29	2	29.549	20	0.24698	1	0.27433

Selenoamino Acid Metabolism	27	1	31.034	20	0.25082	1	0.27433
Pyrimidine Metabolism	57	1	27.273	20	0.28786	1	0.30531
Phenylacetate Metabolism	9	1	27.273	20	0.28786	1	0.30531
Taurine and Hypotaurine Metabolism	12	1	23.077	20	0.33485	1	0.34985
Sphingolipid Metabolism	40	1	12.5	20	0.49177	1	0.49889
Phosphatidylcholine Biosynthesis	14	1	12.5	20	0.49177	1	0.49889
Steroid Biosynthesis	48	1	11.111	20	0.51852	1	0.51852

Table S21. Enrichment of metabolite sets with cisplatin at 20 minutes and 6 hours.

	Total Cmpd	Hits	Statistic Q	Expected Q	Raw p	Holm p	FDR
Phospholipid Biosynthesis	29	2	69.229	20	0.0036725	0.19464	0.049272
Phosphatidylcholine Biosynthesis	14	1	90.21	20	0.0037186	0.19464	0.049272
Oxidation of Branched Chain Fatty Acids	26	1	90.21	20	0.0037186	0.19464	0.049272
Citric Acid Cycle	32	1	90.21	20	0.0037186	0.19464	0.049272
Ketone Body Metabolism	13	2	61.575	20	0.0063399	0.31065	0.067203
Butyrate Metabolism	19	3	60.747	20	0.029873	1	0.26388
Mitochondrial Electron Transport Chain	19	4	55.918	20	0.04417	1	0.29262
Sphingolipid Metabolism	26	4	55.918	20	0.04417	1	0.29262
Carnitine Synthesis	22	1	28.257	20	0.27775	1	0.66411
Phytanic Acid Peroxisomal Oxidation	40	2	25.99	20	0.29957	1	0.66411
Pyruvate Metabolism	47	1	25.904	20	0.30248	1	0.66411
Pyrimidine Metabolism	57	1	23.839	20	0.32582	1	0.66411
Phenylacetate Metabolism	9	1	23.839	20	0.32582	1	0.66411
Retinol Metabolism	35	1	23.12	20	0.33433	1	0.66411
Threonine and 2-Oxobutanoate Degradation	20	1	19.818	20	0.37635	1	0.66411
Lactose Degradation	9	1	18.288	20	0.39764	1	0.66411
Transfer of Acetyl Groups into Mitochondria	22	1	18.288	20	0.39764	1	0.66411
Trehalose Degradation	11	1	18.288	20	0.39764	1	0.66411
Amino Sugar Metabolism	33	3	18.324	20	0.43344	1	0.66411
Valine, Leucine and Isoleucine Degradation	59	3	14.475	20	0.46087	1	0.66411
Propanoate Metabolism	42	2	14.402	20	0.46172	1	0.66411
Glutamate Metabolism	48	3	14.648	20	0.4717	1	0.66411
Ammonia Recycling	31	2	14.577	20	0.47268	1	0.66411
Nicotinate and Nicotinamide Metabolism	35	2	14.577	20	0.47268	1	0.66411
Purine Metabolism	73	2	14.577	20	0.47268	1	0.66411
Beta-Alanine Metabolism	34	1	13.241	20	0.47827	1	0.66411
Cysteine Metabolism	26	1	13.241	20	0.47827	1	0.66411
Glutathione Metabolism	20	1	13.241	20	0.47827	1	0.66411
Lysine Degradation	30	1	13.241	20	0.47827	1	0.66411
Histidine Metabolism	42	1	13.241	20	0.47827	1	0.66411
Folate Metabolism	29	1	13.241	20	0.47827	1	0.66411
Alanine Metabolism	17	1	13.241	20	0.47827	1	0.66411
Tryptophan Metabolism	59	1	13.241	20	0.47827	1	0.66411

Arachidonic Acid Metabolism	67	1	13.241	20	0.47827	1	0.66411
Malate-Aspartate Shuttle	10	1	13.241	20	0.47827	1	0.66411
Tyrosine Metabolism	70	2	13.027	20	0.4832	1	0.66411
Phenylalanine and Tyrosine Metabolism	27	2	13.027	20	0.4832	1	0.66411
Glucose-Alanine Cycle	13	2	14.141	20	0.49443	1	0.66411
Catecholamine Biosynthesis	20	1	11.88	20	0.50346	1	0.66411
Thyroid hormone synthesis	13	1	11.88	20	0.50346	1	0.66411
Warburg Effect	57	6	14.456	20	0.53757	1	0.66411
Inositol Metabolism	30	1	9.5269	20	0.55172	1	0.66411
Pentose Phosphate Pathway	29	1	9.5269	20	0.55172	1	0.66411
Inositol Phosphate Metabolism	24	1	9.5269	20	0.55172	1	0.66411
Urea Cycle	28	3	11.756	20	0.55726	1	0.66411
Aspartate Metabolism	35	3	11.756	20	0.55726	1	0.66411
Gluconeogenesis	33	4	11.659	20	0.60211	1	0.66411
Glycine and Serine Metabolism	59	4	11.053	20	0.60824	1	0.66411
Taurine and Hypotaurine Metabolism	12	1	7.1305	20	0.60897	1	0.66411
Bile Acid Biosynthesis	65	1	7.1305	20	0.60897	1	0.66411
Glycolysis	23	3	10.399	20	0.63736	1	0.66411
Galactose Metabolism	38	3	10.399	20	0.63736	1	0.66411
Nucleotide Sugars Metabolism	20	2	6.9375	20	0.64039	1	0.66411
Starch and Sucrose Metabolism	31	2	6.9375	20	0.64039	1	0.66411
Lactose Synthesis	19	2	11.291	20	0.65885	1	0.67083
Arginine and Proline Metabolism	52	6	9.483	20	0.6933	1	0.6933

Table S22. Enrichment of metabolite sets with CasIIIia at 20 minutes and 6 hours.

	Total Cmpd	Hits	Statistic Q	Expected Q	Raw p	Holm p	FDR
Retinol Metabolism	35	1	99.333	20	1.6696e-05	0.00078471	0.00039235
Valine, Leucine and Isoleucine Degradation	59	1	99.333	20	1.6696e-05	0.00078471	0.00039235
Phospholipid Biosynthesis	29	2	78.734	20	0.00011324	0.0050957	0.00069161
Selenoamino Acid Metabolism	27	2	78.734	20	0.00011324	0.0050957	0.00069161
Biotin Metabolism	8	2	78.734	20	0.00011324	0.0050957	0.00069161
Carnitine Synthesis	22	3	75.887	20	0.00013244	0.0055623	0.00069161
Taurine and Hypotaurine Metabolism	12	3	75.887	20	0.00013244	0.0055623	0.00069161
Bile Acid Biosynthesis	65	3	75.887	20	0.00013244	0.0055623	0.00069161
Catecholamine Biosynthesis	20	3	75.887	20	0.00013244	0.0055623	0.00069161
Thyroid hormone synthesis	13	3	79.972	20	0.00014804	0.0056254	0.00069578
Lysine Degradation	30	5	89.226	20	0.0001898	0.0070226	0.00074736
Glutathione Metabolism	20	2	94.091	20	0.00024854	0.0089475	0.00074736
Alanine Metabolism	17	1	97.407	20	0.00025442	0.0089475	0.00074736
Tryptophan Metabolism	59	1	97.407	20	0.00025442	0.0089475	0.00074736
Arginine and Proline Metabolism	52	1	97.407	20	0.00025442	0.0089475	0.00074736
Beta-Alanine Metabolism	34	1	97.407	20	0.00025442	0.0089475	0.00074736
Cysteine Metabolism	26	3	94.015	20	0.00063106	0.019563	0.0015634
Propanoate Metabolism	42	3	94.015	20	0.00063106	0.019563	0.0015634
Histidine Metabolism	42	2	95.661	20	0.00063202	0.019563	0.0015634
Folate Metabolism	29	1	93.938	20	0.0014068	0.039389	0.0027549
Arachidonic Acid Metabolism	67	1	93.938	20	0.0014068	0.039389	0.0027549
Malate-Aspartate Shuttle	10	1	93.938	20	0.0014068	0.039389	0.0027549
Threonine and 2-Oxobutanoate Degradation	20	1	93.938	20	0.0014068	0.039389	0.0027549
Phenylacetate Metabolism	57	1	93.938	20	0.0014068	0.039389	0.0027549
Pyrimide Metabolism	9	1	0.3771	20	0.908	1	0.99657
Glycine and Serine Metabolism	59	5	3.8889	20	0.91093	1	0.99657
Phosphatidylcholine Biosynthesis	14	1	0.33474	20	0.91331	1	0.99657
Glycolysis	23	1	0.27453	20	0.92148	1	0.99657
Galactose Metabolism	38	1	0.27453	20	0.92148	1	0.99657
Lactose Synthesis	19	1	0.27453	20	0.92148	1	0.99657
Lactose Degradation	9	1	0.27453	20	0.92148	1	0.99657

Transfer of Acetyl Groups into Mitochondria	22	1	0.27453	20	0.92148	1	0.99657
Trehalose Degradation	11	1	0.27453	20	0.92148	1	0.99657
Gluconeogenesis	33	2	0.17484	20	0.94673	1	0.99657
Tyrosine Metabolism	70	2	1.022	20	0.94968	1	0.99657
Phenylalanine and Tyrosine Metabolism	27	2	1.022	20	0.94968	1	0.99657
Amino Sugar Metabolism	33	3	2.3714	20	0.95725	1	0.99657
Urea Cycle	28	4	2.748	20	0.95862	1	0.99657
Aspartate Metabolism	35	3	2.5945	20	0.96175	1	0.99657
Sphingolipid Metabolism	40	2	0.28001	20	0.9795	1	0.99657
Glutamate Metabolism	48	3	0.72002	20	0.97983	1	0.99657
Pyruvate Metabolism	47	1	0.015323	20	0.98143	1	0.99657
Ammonia Recycling	31	2	0.48653	20	0.98182	1	0.99657
Nicotinate and Nicotinamide Metabolism	35	2	0.48653	20	0.98182	1	0.99657
Purine Metabolism	73	2	0.48653	20	0.98182	1	0.99657
Glucose-Alanine Cycle	13	3	0.55524	20	0.98291	1	0.99657
Warburg Effect	57	4	0.28871	20	0.99657	1	0.99657

Table S23. Enrichment of metabolite sets with CasIIgly at 20 min and 6 hours.

**Isotope**	**Relative abundance (%)**
^{63}Cu	69.17
^{65}Cu	30.83

Table S24. Copper isotopes used for ICP-MS determination.

**Sample**	**Cu** (µg de Cu/mL)
01ST20	0.0209
02ST20	0.0440
03ST20	0.0172
**Mean**	**0.0274**
**Standard deviation**	**0.0145**
01ST06	0.0166
02ST06	0.0168
03ST06	0.0136
**Mean**	**0.0157**
**Standard deviation**	**0.0018**
013ia20	0.0152
023ia20	0.0141
033ia20	0.0164
**Mean**	**0.0152**
**Standard deviation**	**0.0012**
013ia06	0.0326
023ia06	0.0316
033ia06	0.0308
**Mean**	**0.0317**
**Standard deviation**	**0.0009**
012gly20	0.0218
022gly20	0.0165
032gly20	0.0237
**Mean**	**0.0207**
**Standard deviation**	**0.0037**
012gly06	0.0271
022gly06	0.0575
032gly06	0.0141
**Mean**	**0.0329**
**Standard deviation**	**0.0222**

Table S25. Copper concentrations in intracellular samples.

Sample	Cu (μg de Cu/mL)
M01ST20	0.0001
M02ST20	-0.0007
M03ST20	0.0040
**Mean**	**0.0011**
**Standard deviation**	**0.0025**
M01ST06	0.0086
M02ST06	-0.0008
M03ST06	0.0151
**Mean**	**0.0076**
**Standard deviation**	**0.0080**
M013ia20	0.1880
M023ia20	0.1738
M033ia20	0.1877
**Mean**	**0.1832**
**Standard deviation**	**0.0081**
M013ia06	0.1636
M023ia06	0.1764
M033ia06	0.0066
**Mean**	**0.1155**
**Standard deviation**	**0.0946**
M012gly20	0.0036
M022gly20	0.0043
M032gly20	0.0128
**Mean**	**0.0069**
**Standard deviation**	**0.0051**
M012gly06	-0.0010
M022gly06	-0.0009
M032gly06	-0.0033
**Mean**	**-0.0017**
**Standard deviation**	**0.0013**

Table S26. Copper concentrations in extracellular samples.
